# Molecular Dynamics Reveals Altered Interactions between Belzutifan and HIF-2 with Natural Variant G323E or Proximal Phosphorylation at T324

**DOI:** 10.1021/acsomega.4c03777

**Published:** 2024-08-26

**Authors:** Vishva Natarajan, Vardhan Satalkar, James C. Gumbart, Matthew Torres

**Affiliations:** † School of Biological Sciences, Georgia Institute of Technology, Atlanta, Georgia 30332, United States; ‡ School of Physics, Georgia Institute of Technology, Atlanta, Georgia 30332, United States

## Abstract

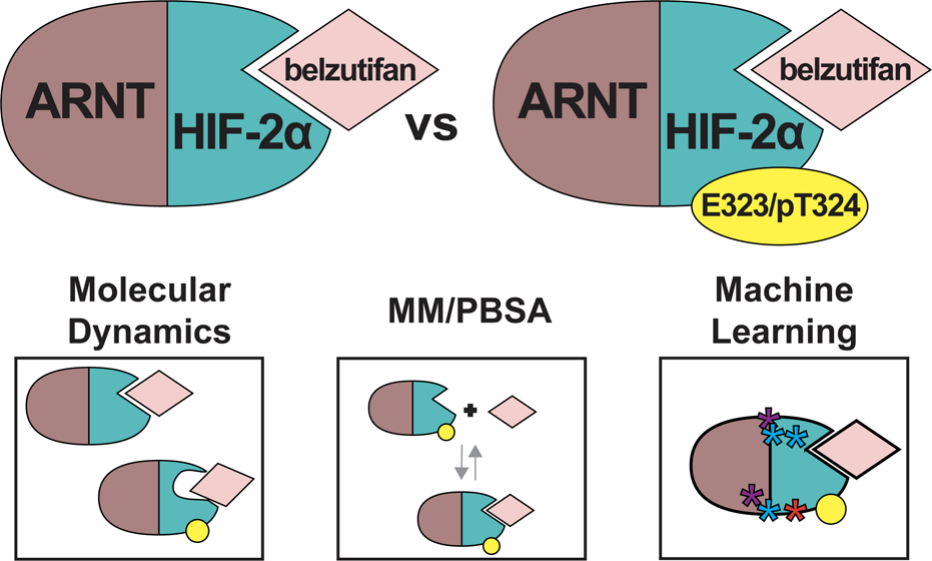

In patients with von-Hippel Lindau (VHL) disease, hypoxia-independent accumulation of HIF-2α leads to increased transcriptional activity of HIF-2α:ARNT that drives cancers such as renal cell carcinoma. Belzutifan, a recently FDA-approved drug, is designed to prevent the transcriptional activity of HIF-2α:ARNT, thereby overcoming the consequences of its unnatural accumulation in VHL-dependent cancers. Emerging evidence suggests that the naturally occurring variant G323E located in the HIF-2α drug binding pocket prevents inhibitory activity of belzutifan analogs, though the mechanism of inhibition remains unclear. Interestingly, proximal phosphorylation at neighboring T324, previously shown to regulate HIF-2 protein interactions, has also been proposed to affect HIF-2 drug binding. Here, we used molecular dynamics (MD) simulations to understand and compare the molecular-level effects of G323E and phospho-T324 (pT324) on the belzutifan bound-HIF-2α:ARNT complex. We find that both G323E and pT324 increase structural flexibility within the drug binding site and reduce the apparent binding affinity for belzutifan. Whereas the effects of G323E are concentrated in the binding pocket Fα helix within the HIF-2α PAS-B domain, pT324 decreased the belzutifan binding affinity and stabilized the HIF-2 heterodimer through an alternate mechanism involving polar interactions between the HIF-2α PAS-B and PAS-A domains. Further analysis via ensemble machine learning uncovered important and distinct interchain residue interactions modified by G323E and pT324. These findings reveal a molecular mechanism of G323E-induced drug resistance and suggest that pT324 may also affect the efficacy of HIF-2 drug binding interactions via allosteric effects.

## 1 Introduction

Transcription factors belonging to the hypoxia inducible factor (HIF) family form heterodimeric structures composed of cellular oxygen-sensing (HIF-α) and nonsensing (HIF-β) dimerization partners that promote the transcription of genes essential for the cellular response to reduced oxygen (hypoxic) conditions.^1−3^ In response to low levels of cellular oxygen, two distinct HIF-α isomers capable of sensing cellular oxygen, HIF-1α and HIF-2α, become upregulated and heterodimerize with the HIF-β counterpart aryl hydrocarbon receptor nuclear translocator (ARNT) through their basic helix–loop–helix (bHLH) and two different period/ARNT/single-minded (PAS-A and PAS-B) domains. Dimerization of HIF-α and ARNT enables DNA binding and the transactivation of genes containing a hypoxia response element (HRE). HIF-2 heterodimers (HIF-2α:ARNT), in particular, drive the expression of erythropoietin (EPO) and vascular endothelial growth factor (VEGF) genes, which promote angiogenesis and cellular proliferation responses that play a critical role in overcoming hypoxic stress.^4−6^

Dysregulation of HIF transcription factors plays a major role in cancer. Both HIF-1α and HIF-2α are overexpressed in most types of human cancers, wherein they promote cell proliferation and vascularization that supports the growth of tumor cells.^7−10^ HIF transcription factors also drive oncogenesis in patients with von Hippel-Lindau (VHL) disease due to a genetic deficiency in the VHL gene that encodes the ubiquitin ligase responsible for promoting the proteasomal degradation of HIF proteins under normoxic conditions.^11^ Owing to the overaccumulation of HIF-2α protein under normoxic conditions, a majority of von Hippel-Lindau (VHL) gene-deficient patients develop several cancers, including clear-cell renal cell carcinoma (ccRCC), CNS hemangioblastoma, and pancreatic neuroendocrine tumors (pNETs).^12^

Due to its prominent role in oncogenesis, multiple HIF-2α antagonists targeting the hydrophobic cavity within the HIF-2α PAS-B domain have been developed to inhibit tumor growth in VHL-associated cancers by disrupting the integrity of HIF-2 heterodimers.^12−16^ These include 0X3, introduced in 2013; PT2399, revealed in 2016; and PT2385, introduced in 2018.^15−17^ Though being the first to enter clinical trials, PT2385 did not receive FDA approval due to extensive *in vivo* glucuronidation in a significant proportion of patients who consequently remained underexposed and received limited clinical benefit.^18^ More recently, the antagonist belzutifan (PT23977) demonstrated high heterodimer disruption potency and improved pharmacokinetic properties that led to its recent approval as a VHL-associated cancer therapeutic by the U.S. Food and Drug Administration.^12^ Based on crystallographic and other biochemical evidence, belzutifan appears to destabilize HIF-2α:ARNT interfacial residues enough to disrupt activity through an allosteric mechanism involving HIF-2α M252 but does not fully dissociate the heterodimer.^19^

While HIF-2 inhibitors have shown high specificity and disruption potency for HIF-2, a naturally acquired mutation of glycine 323 to negatively charged glutamate at position 323 (G323E) within the drug binding pocket renders cancer cells resistant to belzutifan analogs after prolonged treatment.^20−22^ This effect has been studied biochemically, revealing that the G323E variant reduces the ability of belzutifan analogs to dissociate HIF-2α:ARNT and also moderately increases the affinity of HIF-2α for ARNT.^20−22^ Although the introduction of the larger glutamate side chain is consistent with the steric occlusion of belzutifan, a crystal structure for the G323E mutant has not been published, and the precise mechanism of its effect is unknown.

Interestingly, a previously reported biochemical study suggests that PKD1-dependent phosphorylation at the G323-neighboring position, T324, also affects HIF-2 dimerization, raising the question that it too may disrupt drug binding.^23^ However, few studies have evaluated the potential effects of phosphorylation on HIF-2 structure and drug binding amidst mounting evidence that phosphorylation at sites proximal to drug binding pockets can disrupt drug/target activity.^24^ Among well-validated phosphosites in the HIF-2α:ARNT heterodimer, pT324 is the only phosphosite within 12 Å of the drug binding pocket and has been proposed as a site of interest with the potential to interfere with the binding of HIF-2 inhibitors.^24^ Moreover, experimental evidence has shown that pT324 actively hinders interactions between HIF-2α and non-ARNT binding partners while allowing HIF-2α:ARNT heterodimer formation that could counteract HIF-2α antagonism.^23^

Here we utilized a combination of molecular dynamics (MD) simulation, binding free energy calculations, and machine learning to assess the potential effects of G323E or pT324 on HIF-2α:belzutifan and HIF-2α:ARNT interactions. The resulting simulations show that both G323E and pT324 significantly alter the hydrogen bonding network within HIF-2α:ARNT protein complex residues, increase the dynamic flexibility of the PAS-B binding pocket, and reduce the binding affinity of belzutifan while increasing HIF-2α:ARNT dimer stability. Furthermore, key HIF-2α:ARNT interface residues whose interchain interactions differ in response to both G323E and T324 phosphorylation were identified by using an ensemble ML model, many of which are corroborated by previous experimental studies. Taken together, these results provide valuable insight into the dynamic structural effects of both G323E and pT324, which may facilitate the design of improved HIF-2α drugs.

## 2 Materials and Methods

### 2.1 Structure Preparation

Monomeric structures of HIF-2α (AF-P97481-F1) and ARNT (AF-P53762-F1) proteins were obtained from the AlphaFold Database (AFDB)^25,26^ and aligned using the crystal structure of the apo HIF-2α:ARNT heterodimer (PDB 4ZP4 with 2.75 Å resolution) with a root-mean-squared deviation (RMSD) of 1.795 Å and a TM score of 0.85 (Figure S1A–C).^27,28^ The AlphaFold structure was primarily utilized to model the 146 residues missing from the crystal structure (Figure S1D). A belzutifan-bound structure of the HIF-2α:ARNT heterodimer (holo) was then constructed using belzutifan coordinates taken from the crystal structure of the belzutifan-bound HIF-2α:ARNT heterodimer (PDB: 7W80).^19^ A glycine was substituted for a glutamate at position 323 for the G323E mutant belzutifan-bound HIF-2α:ARNT heterodimer (holo-G323E) using PyMOL.^29^ The structural coordinates of the phosphorylated threonine residue (pT324) in belzutifan-bound HIF-2α:ARNT (holo-p) were produced using Vienna-PTM.^30^

### 2.2 Molecular Dynamics

All-atom MD simulations of the holo, holo-G323E, and holo-p complexes were performed using GROMACS version 2021.5.^31^ For all MD simulations, the Amber ff99sb-ILDN force field was used for protein topology generation,^32,33^ whereas AmberTools GAFF parameters were used for ligand belzutifan in all simulations.^34−36^ Protein residue charge states were assigned based on pH 7 using GROMACS preparation tools. Belzutifan atomic charges were calculated using the restrained electrostatic potential (RESP) charge scheme as implemented in AMBER.^35,37^ For each simulation, the complex was centered in a dodecahedron box with a 10 Å distance between the protein surface and box edge. The protein complex was then solvated using the TIP3P explicit water model, and Na^+^ ions were added to render the system electroneutral.^38^

Energy minimization was performed by restraining protein heavy atoms and applying the steepest descent algorithm with step size 0.01 until the potential energy converged with a maximum force of no greater than 1000 kJ mol^–1^ nm^–1^. After energy minimization, the ligand-restrained equilibration was performed at a temperature of 300 K under the isothermal–isochoric (NVT) ensemble for 100 ps with a 2 fs time step. The equilibration was continued under the isothermal–isobaric (NPT) ensemble for 100 ps using a time step of 2 fs and pressure coupling using a Berendsen barostat under periodic boundary conditions. For both NVT and NPT simulations, the particle mesh Ewald (PME) method was used for long-range electrostatics with a short-range cutoff value of 12 Å. The LINCS algorithm was used for holonomic constraints involving bonds to hydrogen. We confirmed the convergence of the potential energy, density, pressure, and temperature following the equilibration (Figure S2). Finally, all positional restraints were removed prior to production runs at 300 K.^39^ Overall, five production replicates of 300 ns each for the holo, holo-G323E, and holo-p states for a total of 15 simulations and 4.5 μs of simulation time were collected.

### 2.3 Analysis of Geometric Properties

To assess the movement of both protein and ligand with respect to the structure with the lowest energy after minimization, the RMSD of both the protein backbone and belzutifan ligand was computed after performing least-squares fitting to the protein backbone. To analyze structural flexibility, the Cα root mean squared fluctuations (Cα-RMSF) were calculated on a 1,250 ns trajectory formed from concatenating the last 250 ns of each of 5 holo, holo-G323E, or holo-p trajectories. The initial equilibrated structure from the first holo/holo-G323E/holo-p replicate was used as the reference structure for RMSF. For the DSSP analysis and secondary structure annotations, 25,000 evenly spaced frames were sampled from the concatenated holo, holo-G323E, or holo-p trajectory. For each residue, the percentage of frames assigned to each DSSP code was computed using MDTraj^40^ and custom Python scripts.^41,42^ Hydrogen bond (H-bond) occupancy calculations were performed using VMD.^43^ The donor–acceptor distance was specified as 3.5 Å, the hydrogen donor–acceptor angle cutoff was 30°, and only polar atoms were considered. To analyze the H-bonding between belzutifan and HIF-2α or G323E/pT324 and HIF-2α, the final 250 ns of each replicate was used. For the analysis of belzutifan-water-HIF-2α H-bond networks, 250 evenly spaced frames from the last 250 ns of each replicate were used.

### 2.4 Binding Free Energy Calculation and Per-Residue Decomposition

The ligand–protein interaction energy between belzutifan and the HIF-2α:ARNT heterodimer and the protein–protein binding free energy between HIF-2α and ARNT were computed using the molecular mechanics Poisson–Boltzmann surface area (MM/PBSA) method using the g_mmpbsa tool.^44,45^ To perform binding free energy analysis, 500 uniformly distributed conformations were evenly sampled from the last 250 ns of each MD trajectory. The total binding free energy (Δ*G*_Total_) values were reported along with the following three energy components: (1) the molecular mechanics energy (Δ*E*_MM_); (2) the polar solvation energy (Δ*G*_Polar_)_;_ and (3) the nonpolar solvation energy (Δ*G*_Nonpolar_). Further energy decomposition analysis was performed to obtain the per-residue contributions of each energy component.

### 2.5 Statistical Methods

The one-tailed Mann–Whitney U test was used to assess the statistical significance of each structural and energetic perturbation observed in holo-G323E or holo-p relative to holo. A p value of less than 0.05 was considered to be statistically significant.

### 2.6 Analysis of Interchain Interactions Using Ensemble ML Tools

An ensemble machine learning methodology developed by Pavlova et al. was employed to determine the most important residues distinguishing holo-G323E from holo and holo-p from holo heterodimer interactions at four interchain HIF-2α:ARNT interfaces and two intrachain HIF-2α interfaces.^28,46^ To begin, 5,000 evenly spaced frames from the final 250 ns of each of 5 holo, holo-G323E, and holo-p trajectories were obtained. To prepare input features, interfacial residue pairs with a contact distance of less than 10 Å in at least one frame from either all holo and holo-G323E or all holo and holo-p MD simulation replicates were identified.

Regarding a comparison of interfaces between holo and holo-G323E, HIF-2α bHLH:ARNT bHLH (interface 1) had 527 features; HIF-2α PASA:ARNT PASA (interface 2) had 922 features, HIF-2α PASB:ARNT PASA (interface 3) had 265 features, HIF-2α PASB:ARNT PASB (interface 4) had 473 features; HIF-2α PASB:PASA (interface 5) had 395 features; and HIF-2α PASA:bHLH (interface 6) had 576 features. Regarding a comparison of interfaces between holo and holo-p, interface 1 had 537 features, interface 2 had 947 features, interface 3 had 243 features, interface 4 had 518 features, interface 5 had 383 features, and interface 6 had 581 features.

After residue pair input features were prepared, a matrix where every column was a residue pair and every row was a frame containing the inverse of coresidue contact distance was produced for each separate interface. All values were adjusted so that each feature had mean of 0 and a standard deviation of 1. To obtain important residues, the following procedure was repeated 20 times: removal of highly correlated features (correlation >0.9) and then training of logistic regression (LR), random forest (RF), and multilayer perceptron (MLP) models to predict each frame as belonging to either G323E-HIF-2α/pT324-HIF-2α or HIF-2α with an accuracy of 1.0. The importance of individual residues was computed by summing the importance of a residue over all corresponding residue pairs. The removal of highly correlated features was shuffled in each iteration, and the importance scores were averaged over all 20 iterations. All machine learning models were implemented using the scikit-learn, NumPy, and pandas Python libraries.

The classification of important residues was performed using the following criteria: the residues with importance score >0.8 in at least one of the ML models were considered to be category 1. Those residues with an absolute difference in Δ*G*_Total_ ≥ 2 kJ/mol between holo and holo-G323E/holo-p or an absolute difference in ΔRMSF of ≥1 Å (≥3 Å for disordered regions) were classified as category 2. Finally, those residues meeting all three criteria were considered to be category 3.

## 3 Results

### 3.1 G323E and pT324 Increase the Flexibility of the Belzutifan Binding Pocket in HIF-2α

Prior evidence suggests that the negatively charged G323E mutation proximal to the HIF-2α drug binding pocket hinders the ability of the HIF-2α PAS-B domain to interact with inhibitors.^21,22^ To further investigate the structural and drug binding effects of G323E and pT324, we compared 5 independent 300 ns MD simulations for each of three different HIF-2α:ARNT:belzutifan structures: unmodified (holo), glutamate substituted for glycine at position 323 (holo-G323E), and the phosphorylated at T324 (holo-p). RMSD convergence occurred at 50 ns in each replicate, suggesting that the simulations effectively captured equilibrium states (Figure S3).

**Figure 1:**
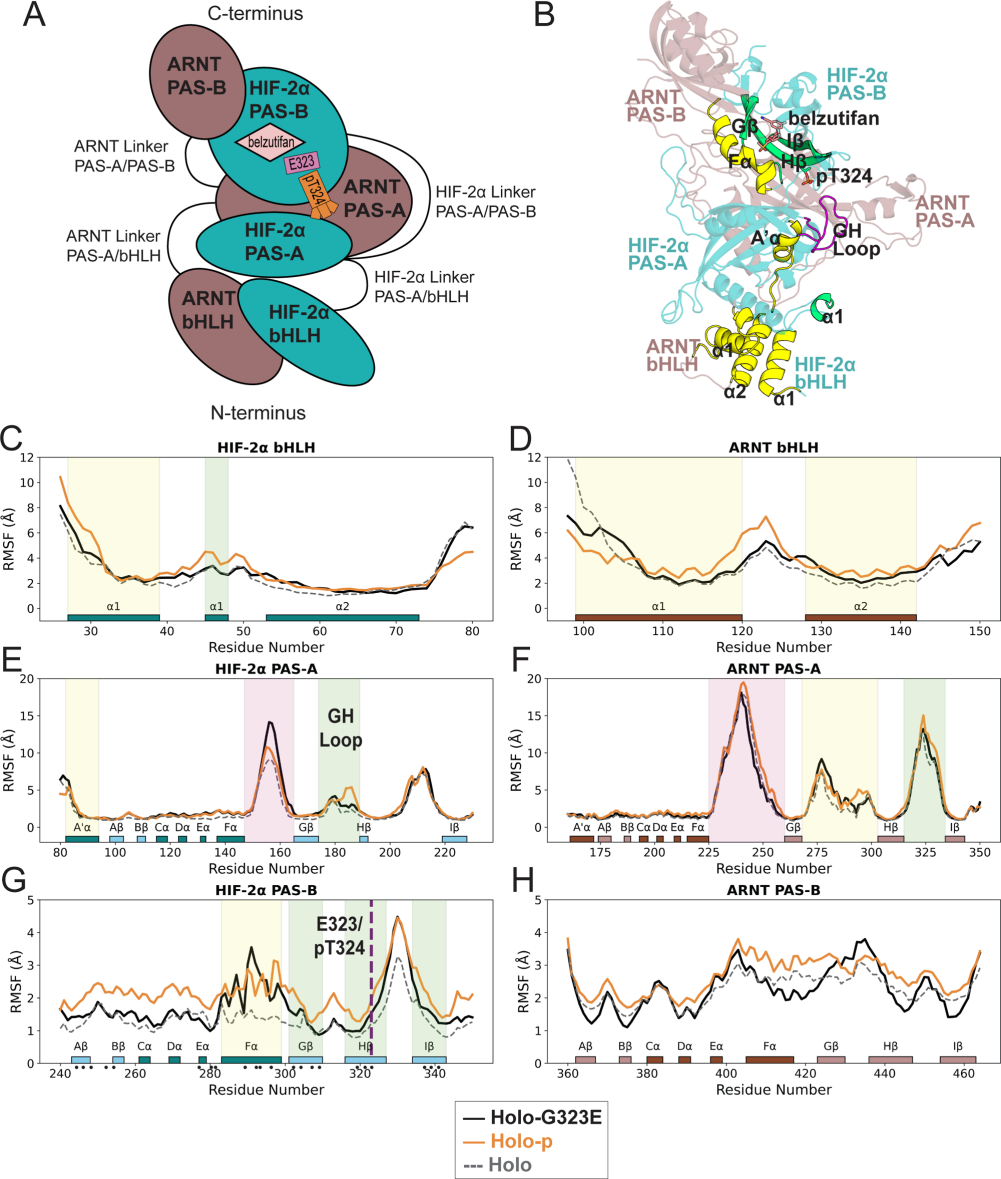
Domains of the HIF-2α:ARNT:belzutifan complex and a comparison of RMSF for all Cα atoms in holo-, holo-G323E, and holo-p simulations. (A) Schematic diagram of HIF-2α domain architecture including the relative locations of the belzutifan binding pocket, the G323E mutation, and threonine phosphorylation site pT324. HIF-2α is shown in a teal color, while ARNT is depicted in a brown color. (B) Representative holo-p structure highlighting ordered regions with threshold-surpassing RMSF differences observed for both holo-G323E and holo-p relative to holo (yellow) or specific to holo-p alone (green). (C–H) Structural Cα-RMSF (Å) for the bHLH domain of HIF-2α and ARNT, PAS-A domain of HIF-2α and ARNT, and PAS-B domain of HIF-2α and ARNT in holo (in gray dotted lines), holo-G323E (in black solid lines), and holo-p (in orange solid lines) simulations. DSSP-assigned secondary structures are represented as colored boxes, with a darker hue representing the α helix and a lighter hue representing beta sheets. The approximate location of both E323 and pT324 on Hβ of HIF-2α PAS-B is indicated by a vertical dashed line. The threshold difference in RMSF was considered to be at least 1 Å for structured regions and 3 Å for unstructured regions. Regions with at least one residue having a notably different RMSF in both holo-G323E and holo-p relative to holo (yellow shaded boxes), unique to holo-G323E (pink shaded box), or unique to holo-p (green shaded boxes). Black dots indicate residue side- and main-chain contacts with belzutifan (within a 4 Å distance).

To assess whether HIF-2α:ARNT domains were affected by G323E or pT324, we computed the Cα-RMSF, a measure widely regarded to indicate structural flexibility, for each residue in the heterodimer (Figure 1A,B). We considered a region to have notable structural fluctuations if one or more residues had at least a 1 or 3 Å absolute difference in RMSF in the simulations for holo-G323E or holo-p relative to holo for ordered or disordered regions, respectively (Figure 1C–H, yellow/green/pink panels). Notably, RMSF values ranging from −4.9 to +5.0 Å were observed in structured and unstructured regions spanning all three domains of HIF-2α and exclusively in the bHLH and PAS-A domains of ARNT (Tables S1 and S2). Overall, the RMSF results point to a broad range of structural changes that were specific to mutation, phosphorylation, or either state.

Notably, both G323E and pT324 exhibit dynamic effects observed in all but the PAS-B domain of ARNT but predominantly in HIF-2α (Figure 1C–H). Both mutation and phosphorylation promoted unique fluctuations in neighboring loop structures between the Fα-Gβ or Gβ-Hβ ordered regions of HIF-2α PAS-A, suggesting an influence on interdomain dynamics of disordered structures (Figure 1E). In contrast, the dynamic fluctuations of HIF-2α PAS-B were observed only for ordered regions that make up the belzutifan binding pocket as defined by the recent crystal structure from Ren et al.^19^ PAS-B regions were influenced uniquely by pT324 for beta strands G-I as well as a shared effect with G323E in the Fα helix (Figure 1G). Similar evidence of a shared dynamic effect for G323E and pT324 was limited to the α1 and A′α of the respective HIF-2α bHLH and PAS-A domains as well as the α1 and α2 helices of ARNT bHLH (Figure 1D).

To further evaluate whether the observed differences in RMSF corresponded to increased disorder in secondary structures comprising the belzutifan-bound binding pocket, we compared the distribution of per-residue secondary structure assignments for holo, holo-G323E, and holo-p domain residues in the PAS-B domain as annotated using the DSSP algorithm.^41^ For each residue, we computed the average percentage of frames assigned to each DSSP code over 1,250 frames sampled from the concatenated trajectories.

**Figure 2:**
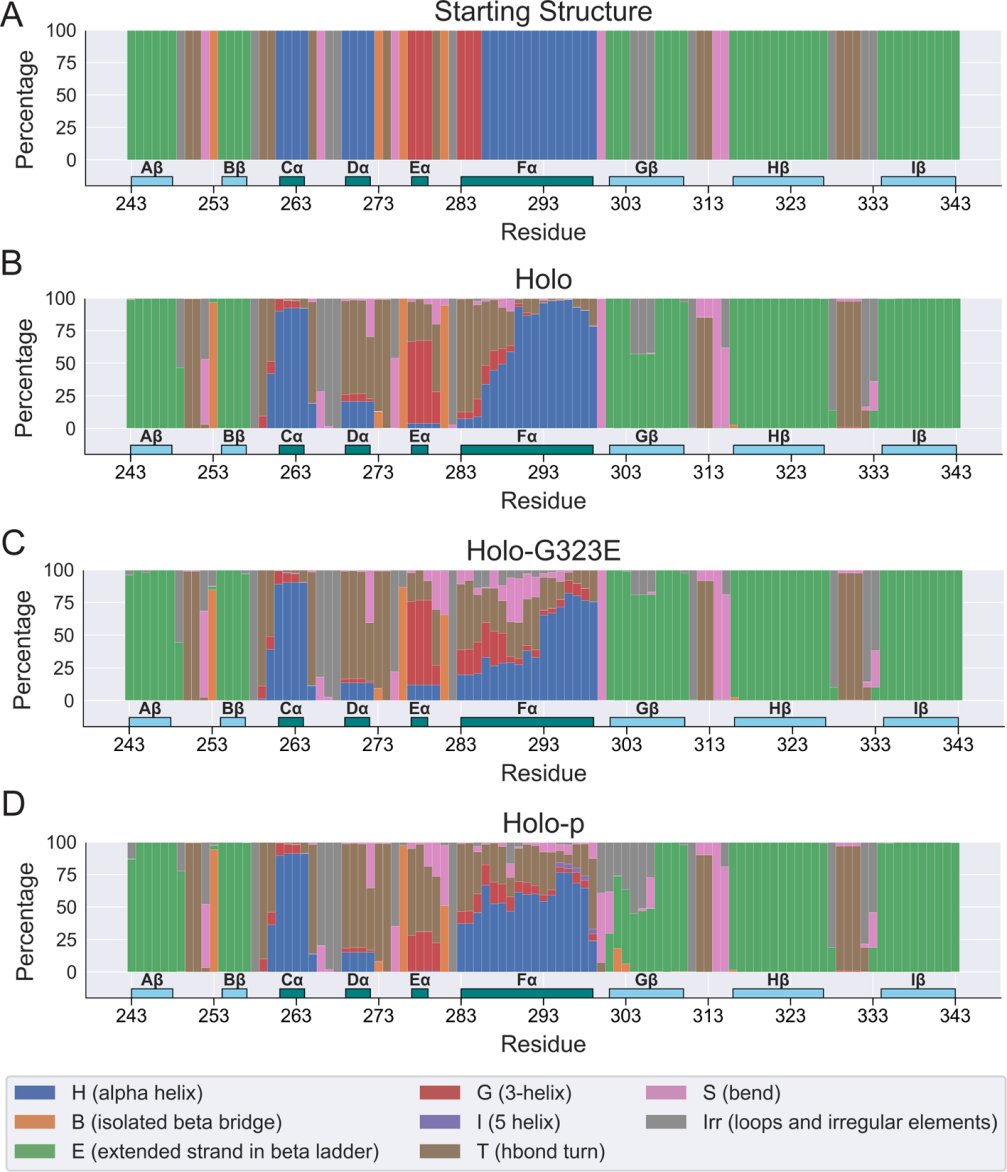
Flexibility in the secondary structure of PAS-B domains. (A) The secondary structure annotations (in %) for the initial AlphaFold structure are shown for PAS-B residues. (B–D) The average secondary structure annotations (in %) for the simulated holo, holo-G323E, and holo-p structures are shown for PAS-B residues for 1,250 frames evenly sampled from MD trajectories of length 1,250 ns concatenated using the last 250 ns of 5 replicates. For secondary structure annotations, light-blue boxes represent β-sheets while dark-teal boxes represent α-helices of the HIF-2α PAS-B domain.

Discrete differences in the DSSP annotation between holo, holo-G323E, and holo-p were evident across the complex but most notably for HIF-2α PAS-B, which contains the belzutifan binding pocket (Figure 2A–C). The largest of these differences was observed for the Fα helix and its neighboring regions. In Fα, which contains the key H293 residue required to stabilize belzutifan, a greater number of N-terminal residues up to H293 were distinctly more turn-like in holo-G323E compared to holo, while the C-terminal residues had only a slight decrease in α-helical character (Figure 1B). However, this distribution in average secondary structure was not seen in the holo-p state, which was more helical overall with a more uniform distribution of the α helix and turn-like character from N to C (Figure 2C).

In the neighboring Eα helix, a distinct shift from more helical to more turn-like character was evident for holo-p but not matched by holo-G323E. Similarly, we found a reduction in the average percentage of β strand residues in the Fα-neighboring Gβ sheet in holo-p compared to both the holo- and holo-G323E states, suggesting a decrease of β character in response to phosphorylation but not G323E mutation. The secondary structures of most of the other regions in HIF-2α PAS-B were comparatively unperturbed.

Similar to PAS-B, the bHLH domain of HIF-2α, which showed increased flexibility in a single S28 residue in holo-G323E and several residues in holo-p, also exhibited distinct differences in the average secondary structure compared with holo (Table S1). These changes were exclusive to helix α1, which had a more pronounced N-to-C distribution in turn versus helical character in holo-p that was not as pronounced in either the holo- or holo-G323E state (Figure S4). A nearly identical trend was found for helix α1 in the bHLH domain of ARNT for both holo-G323E and holo-p compared to holo (Figure S5). Phosphorylation-dependent changes in the secondary structure were not observed to a significant degree for the HIF-2α PAS-A domains (Figure S6).

In summary, these data demonstrate that both G323E and pT324 increase the flexibility of ordered and disordered regions within the bHLH and PAS domains. Most notably, both proteoforms demonstrated increased disorder of secondary structure regions not only directly interacting with belzutifan but also distant from the drug binding pocket and proximal to the DNA-binding head, with pT324 demonstrating relatively uniform effects.

### 3.2 Analysis of E323 and pT324 Interactions

To understand the plausible underlying mechanism by which G323E and pT324 induce structural flexibility and disorder in the HIF-2α:ARNT complex, we analyzed the hydrogen bond (H-bond) occupancies of H-bond donors to E323 and threonine phosphate as acceptors. In this case, we focused on H-bonds that had a minimum of 10% H-bond occupancy in at least one of the holo or holo-G323E/holo-p replicates.

**Figure 3:**
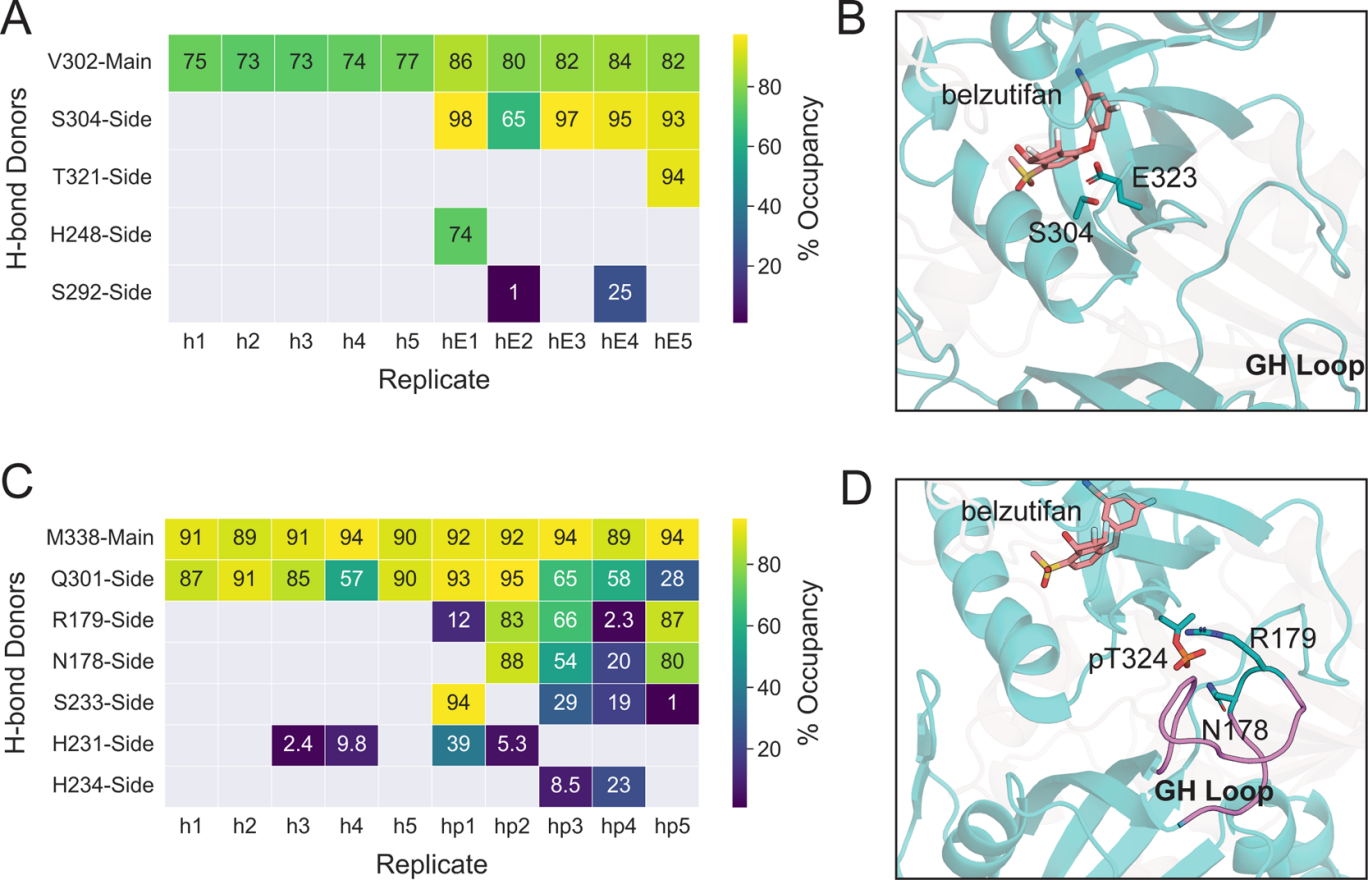
Analysis of H-bonding interactions of G/E323 and T/pT324 as H-bond acceptors with key H-bond donors. (A) H-bond occupancy of HIF-2α residues with G/E323 shown in both holo (h1–h5) and holo-G323E (hE1-hE5) replicates, respectively. Values *<*1 are grayed out. (B) Key H-bond interaction between G323E and S304 shown using a representative frame from holo-G323E replicate 1. (C) H-bond occupancy of HIF-2α residues with T324 and pT324 shown in both holo (h1–h5) and holo-p (hp1–hp5) replicates, respectively. (D) Key H-bond interactions between pT324 and GH loop residues N178 and R179 shown using the final frame from holo-p replicate 2.

Whereas V303 remained an H-bond donor to the main chain of residue 323 for all holo- and holo-G323E replicates, the substitution from glycine to glutamate at position 323 resulted in a new H-bond donated by S304, located on the binding pocket Gβ strand, that was observed across all holo-G323E replicates (Figure 3A,B). T324 phosphorylation similarly introduced several novel H-bonding interactions. For both T324 and pT324 H-bond acceptors, amino acid residues M338, Q301, and H231 acted as the key H-bond donors to either the main or side chain of the threonine phosphate group (Figure 3C). In the holo-p replicates, pT324 accepted H-bonds from H234 and S233 located on the disordered PASB–PASA linker of HIF-2α. Most notably, unlike T324, pT324 accepted H-bonds from N178 and R179 on the GH loop located at the PAS-A/PAS-B interface (see Figure 1E), indicating the effects of pT324 on conformational changes outside the PAS-B domain (Figure 3C,D).

Upon further investigation into the dynamics of pT324/GH loop interactions, we observed a repetitive tugging motion that was facilitated by H-bonding between pT324 and N178/R179 (Supporting Information Movie S1). This caused the GH loop to be frequently pulled toward HIF-2α PAS-B, revealing a significant difference between holo- and holo-p simulations (Figure 3D). In contrast, a similar tugging motion between these residues did not occur frequently in any of the five holo simulations (Supporting Information Movie S2). This was supported by the significantly larger distances observed between the corresponding donor–acceptor pairs in holo- versus holo-p simulations. For instance, the average distance between H-bond donor N178 and the H-acceptor T324/pT324 was 7.84 Å in the holo compared to 3.36 Å in holo-p trials (*p* value = 0.004) (Figures S7 and S8). Similarly, the average distance between H-bond donor R179 and H-bond acceptor T324/pT324 was 11.79 Å in holo relative to the 4.42 Å in holo-p trials (*p* value = 0.004) (Figures S7 and S8).

Finally, in addition to the introduction of new H-bonds, we found a statistically higher HIF-2α PAS-B domain average solvent-accessible surface area (SASA) of 66.71 ± 0.48 nm^2^ in the holo-G323E state and 66.94 ± 0.68 nm^2^ in the holo-p state compared to the 64.61 ± 0.73 nm^2^ in the holo state (*p* = 0.004 for both), suggesting that both G323E and pT324 decrease the compaction of the PAS-B drug-binding domain to a similar extent. Taken together, these results indicate novel H-bonding interactions and PAS-B conformational changes for the holo-G323E and holo-p states associated with an increase in the flexibility of the drug binding pocket. Notably, the pattern of H-bonding to pT324 suggests a plausible mechanism, whereby phosphorylation increases the flexibility of the PAS-B domain through dynamic movement of the GH loop.

### 3.3 Identification of the Key Domains Using Protein–Protein BFE Decomposition Analysis

Following the observation of structural perturbation caused by G323E and pT324, we estimated the protein–protein binding free energy (BFE) between HIF-2α and ARNT monomeric units for holo-, holo-G323E, and holo-p MD simulations using MM/PBSA (see Methods). This analysis was performed to determine the role of E323 and phosphorylation on heterodimer stability in the drug-bound state.

The mean total BFE was found to be lower in both holo-G323E (−203.6 kJ/mol) and holo-p (−237.2 kJ/mol; p = 0.016) compared to the holo state, demonstrating that both G323E and pT324 stabilize the HIF-2α:ARNT heterodimer and that the latter had a more pronounced effect (Table 1). Per-residue BFE decomposition of each domain further revealed the ARNT bHLH domain as having the largest stabilizing effect in both holo-G323E (ΔBFE = −83.3 kJ/mol) and holo-p (ΔBFE = −160.0 kJ/mol) compared to the holo state (Tables S3 and S4). For holo-G323E, this was followed by the HIF-2α PAS-A (ΔBFE = −44.5 kJ/mol) and ARNT PAS-A (ΔBFE = −26.6 kJ/mol) domains, whereas for holo-p, the HIF-2α PAS-B (ΔBFE = −77.3 kJ/mol) and PAS-A (ΔBFE = −50.4 kJ/mol) domains had the next highest stabilizing effects (Tables S3 and S4). In the holo-p state, we also noted a relatively large stabilizing effect (ΔBFE = −20.8 kJ/mol) of the GH loop involved in the tugging motion with pT324 (Table S4, Figure 3).

**Table 1:** Total Binding Free Energy Values (kJ/mol) for the HIF-2α:ARNT Protein–Protein Interactions Obtained Using Five Holo, Holo-G323e, and Holo-p Replicates, with the Mean ± Standard Deviation of the Five Values

	Protein–Protein Binding Free Energy (kJ/mol)		
Replicate	Holo	Holo-G323e	Holo-p
1	–1228.0 ± 193.7	–1442.5 ± 204.5	–1506.7 ± 178.6
2	–1142.0 ± 193.0	–1721.9 ± 207.0	–1500.3 ± 185.8
3	–1456.0 ± 221.9	–1273.8 ± 207.8	–1341.7 ± 176.4
4	–1305.0 ± 246.9	–1190.5 ± 217.4	–1523.5 ± 229.4
5	–901.2 ± 171.8	–1421.1 ± 166.8	–1346.0 ± 203.9
Mean	–1206.4 ± 206.0	–1410.0 ± 203.2	–1443.6 ± 91.5
Δ		–203.6	–237.2

An analysis of the top 20 residues from HIF-2α and ARNT that contribute the most to relative stabilization of the holo-G323E/holo-p versus the holo state further revealed an enrichment of charged residues (D, K, E, and R) in HIF-2α and (K and R) in ARNT (Tables S5–S8). In most of these cases, the largest decreases in Δ*G*_Total_ were attributable to the Δ*E*_MM_ component, suggesting that both G323E and pT324 may stabilize drug-bound HIF-2α:ARNT by facilitating highly stabilizing protein–protein electrostatic interactions (Tables S5–S8).

### 3.4 Identification of Important Interfacial Remains Using Machine Learning (ML) Tools

**Figure 4:**
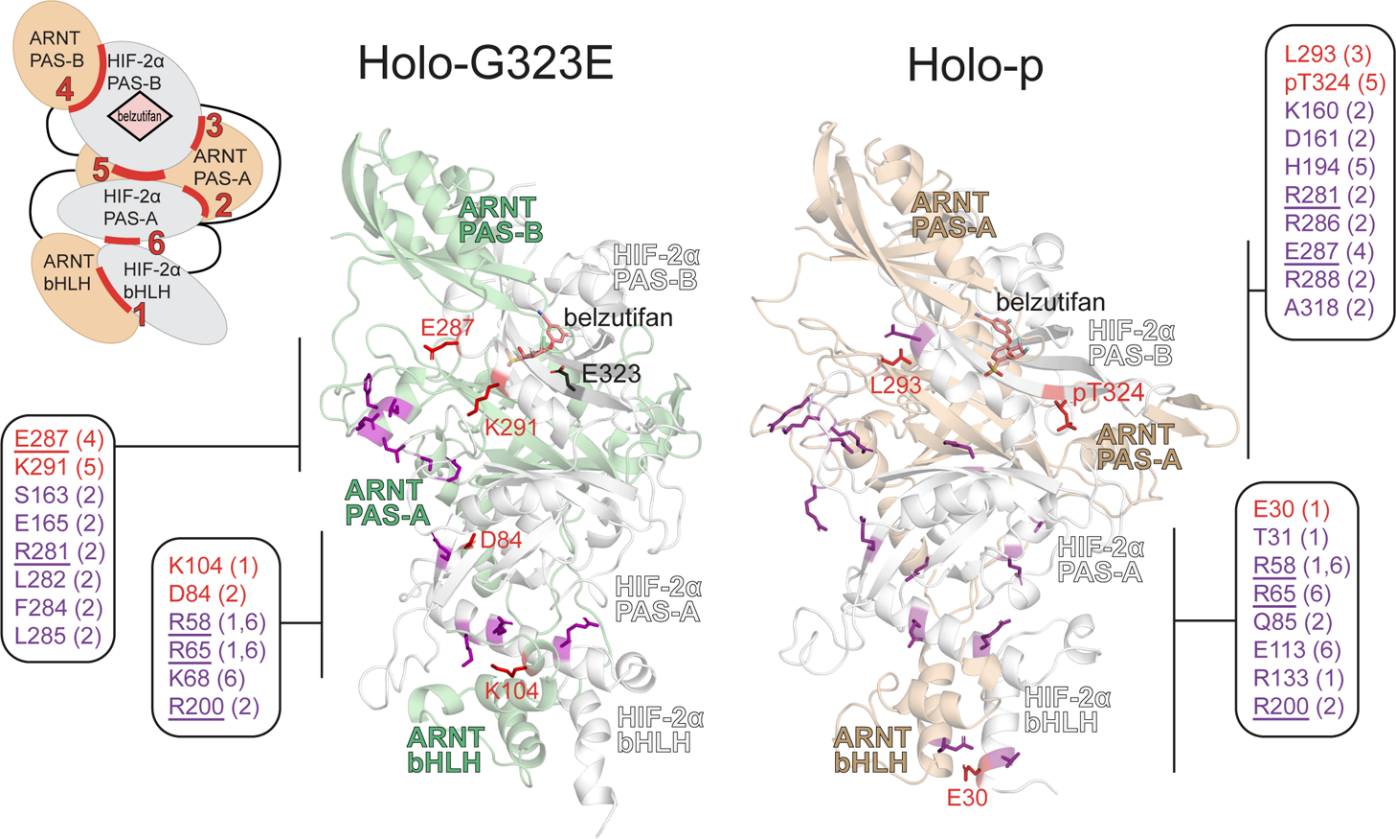
ML results combined with Δ*G*_Total_ and ΔRMSF. Schematic of HIF-2α:ARNT domain architecture with six interfaces labeled. Representative frames from holo-G323E and holo-p replicates are shown, with category 2 (purple) and category 3 (red) residues indicated. Category 2 residues have either absolute Δ*G*_Total_ ≥ 2 kJ/mol or ΔRMSF ≥ 1 Å (≥3 Å for disordered regions) in addition to having importance >0.8 in at least one ML model. Category 3 residues meeting all three criteria for importance are labeled in the structures. Boxes listing the category 2 and 3 residues for each region in the structure are included, with the interface number in parentheses and the residues of at least category 2 in both holo-G323E and holo-p underlined.

Given that MD trajectories often consist of multidimensional residue interaction data, several ML tools have been increasingly developed to pinpoint important interfacial residue interactions.^46−49^ To elucidate important residues involved in fine-grained interactions distinguishing G323E-mutated HIF-2α:ARNT or pT324-HIF-2α:ARNT from the unphosphorylated, wild-type heterodimer, an ensemble of three ML models was utilized to analyze six interfaces determined previously to be critical to the stability of the HIF-2α:ARNT complex.^28^ Based on the ML-generated residue importance score, several interfacial residues were identified as important (Figure 4). We further classified these into three categories based on whether the residue also affected either the protein–protein binding free energy (Δ*G*_Total_) or RMSF (category 2), neither (category 1), or both (category 3) (see Methods).

Notable similarities were observed between residues classified as category 2 or 3 for both holo-G323E and holo-p. In both states, residues R58 and R65, located in the HIF-2α bHLH domain, were classified as category 2 for interchain interface 1 and intrachain interface 6. At interface 2 between the PAS-A domains of both proteins, HIF-2α residue R200 and ARNT residue R281 were classified as category 2 for both states. Between the PAS-B domains comprising interface 4, HIF-2α residue E287, which had a dimer-stabilizing effect, was classified as categories 3 and 2 in holo-G323E and holo-p, respectively (Figures S9 and S10, Tables S9 and S10).

While G/E323 was not an important interfacial residue, pT324, located at interface 5, was classified as a category 3 residue, demonstrating both a high dimer-stabilizing effect (ΔΔ*G*_Total_ = −94.0 kJ/mol) and increased flexibility (ΔRMSF = 1.055) (Table S10). Interface 5 also contained residue F169, which was classified as category 1 in both holo-G323E and holo-p comparisons and was previously demonstrated through co-immunoprecipitation (co-IP) to be essential to dimer stability. Two additional interface 5 residues, H194 (category 2) and V192 (category 1), were important only in holo-p and were also previously highlighted by co-IP studies (Figure S9 and Table S10).

While residues of category 3 were found at interfaces 1 and 5 for both holo-G323E and holo-p, only holo-G323E contained a category 3 residue at interface 2, namely D84 (ΔΔ*G*_Total_ = −10.5 kJ/mol), and only holo-p contained a category 3 residue at interface 3, L293 (ΔΔ*G*_Total_ = 4.7 kJ/mol) (Figure S9 and Table S6). Overall, the highest occurrence of residues 2 and 3 was observed at interfaces 1, 2, and 6 for both proteoforms, suggesting that G323E and pT324 selectively modulate the interfaces formed by the PAS-A and bHLH domains of both HIF-2α and ARNT. The key interfacial residues identified at each interface suggested overlapping but distinct allosteric effects of G323E and pT324 on the dynamics of belzutifan-bound HIF-2α:ARNT.

### 3.5 Effects of G323E and pT324 on Belzutifan Binding to HIF-2α

Given that G323E is established to disrupt the binding of belzutifan,^20−22^ we next investigated how phosphorylation may affect the binding of belzutifan to HIF-2α by comparing the geometric and energetic properties of belzutifan in the holo, holo-G323E, and holo-p states. An analysis of ligand flexibility using the RMSD for belzutifan revealed that compared to the holo (2.0 ± 0.2 Å) simulations, the holo-G323E (4.1 ± 0.9 Å; p = 0.004) and holo-p (2.7 ± 0.7 Å; p = 0.075) states exhibit greater variability in belzutifan movement within the binding pocket, with the variability of holo-G323E being statistically greater than that of holo-p (p = 0.027) (Figure S11).

**Figure 5:**
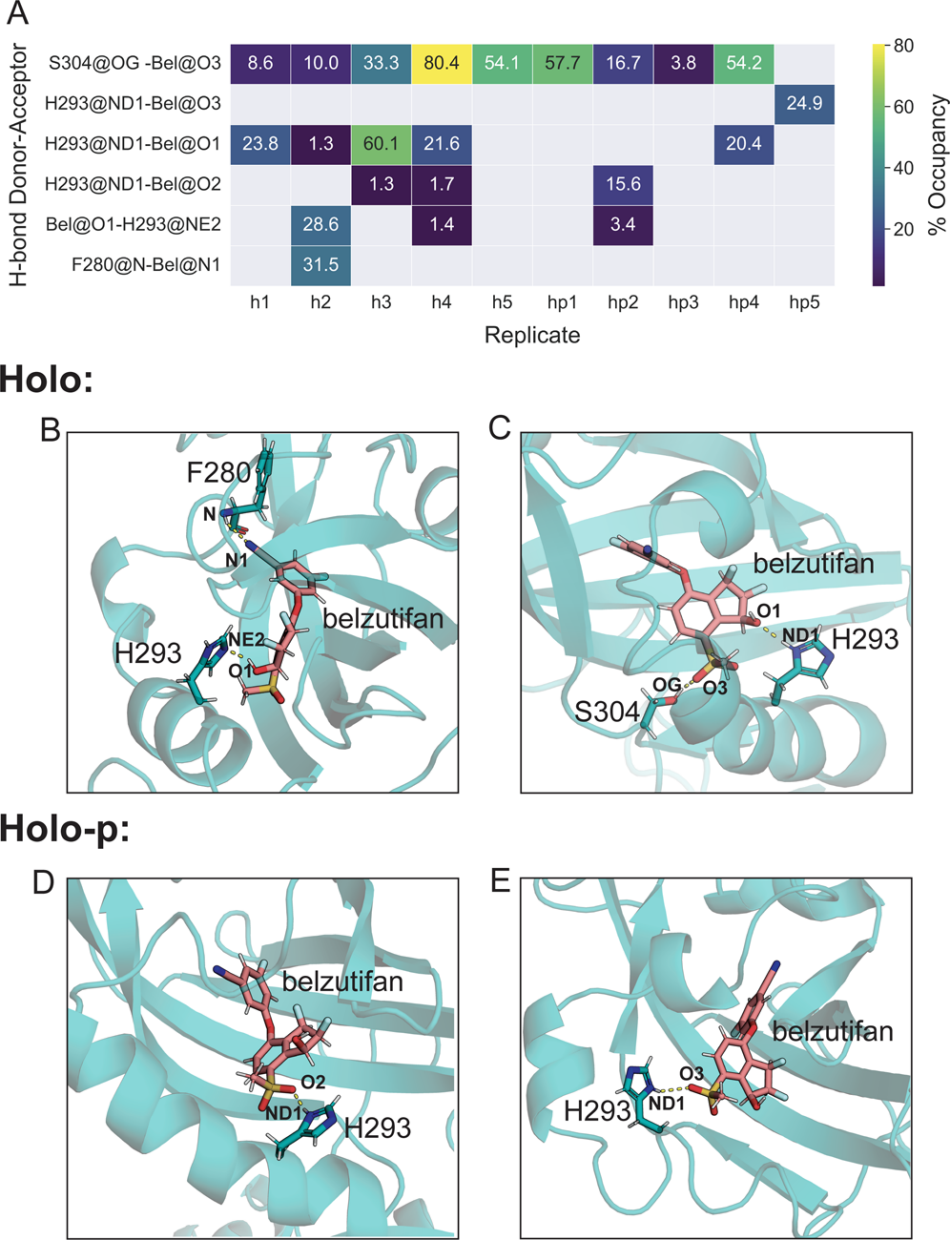
H-bond occupancies for the belzutifan ligand in the HIF-2α PAS-B binding pocket. (A) Heat map showing H-bond occupancies for all donor–acceptor pairs with at least 10% occupancy in at least one holo or holo-p replicate. (B) Representative holo-p structure from holo replicate 2 showing H-bond of F280 N and H293 NE2 with belzutifan N1 and O1, respectively. (C) Representative holo-p structure from holo replicate 3 showing the H-bond of S304 OG and H293 O3 with belzutifan O3 and O1, respectively. (D) Representative holo-p structure from holo-p replicate 4 showing the H-bond of the H293 ND1 atom with the O2 of belzutifan. (E) Representative holo-p structure from holo-p replicate 5 showing the H-bond of H293 ND1 with the O3 of belzutifan.

Next, the H-bond network between belzutifan and nearby residues in the PAS-B binding pocket was analyzed to better understand the ligand-binding interactions disrupted by G323E and the possible effects of phosphorylation on the binding pose of the ligand. Strikingly, H-bond occupancy results revealed that HIF-2α residues H293 and S304, which consistently formed H-bonds with belzutifan atoms in both the holo- and holo-p states, did not H-bond to any belzutifan atoms in the holo-G323E state, confirming that the mutation disrupts key interactions between HIF-2α and belzutifan (Figure S12). Though H-bonds between H293/S304 and belzutifan were observed for both holo and holo-p, there were distinct differences in the occupancies and donor–acceptor atom pairs. In particular, S304 had an H-bond occupancy greater than 10% in all holo replicates via direct coordination with belzutifan, whereas the H-bond occupancy of S304 was less than 10% in two holo-p replicates (Figure 5). Furthermore, interactions between H293 and belzutifan differed between the holo- and holo-p states. For instance, in the holo simulations, three out of five replicates showed that the ND1 atom of H293 donated an H-bond to the O1 atom of belzutifan. In contrast, only one out of five holo-p replicates showed the same bond. A similar disruption of H-bonding between H293 and belzutifan for holo-p was observed for the HIE form of H293 (Figure S13).

Given that structural water molecules have been shown to affect HIF-2α drug-binding interactions,^16,50−52^ we also scanned for hydrogen bonds formed between water, HIF-2α PAS-B domain residues, and belzutifan. We discovered a greater number of distinct structural water H-bond networks for holo-G323E and holo-p states compared with the holo state (Table S12).

Like holo-G323E, the significant changes in the ligand pose of belzutifan observed between the holo- and holo-p states suggested a possible change in ligand affinity in response to phosphorylation. To evaluate this possibility, we compared the total BFE of belzutifan between the holo, holo-G323E, and holo-p states by calculating the difference between holo and holo-G323E/holo-p after summing molecular mechanics, polar, and nonpolar BFE components. Compared to holo (−124.4 kJ/mol) states, the mean total BFE was found to be 51.7 kJ/mol greater in holo-G323E (−72.7 kJ/mol; *p* = 0.004) and 23.4 kJ/mol greater in holo-p (−101.0 kJ/mol; *p* = 0.028), suggesting that pT324 destabilizes belzutifan in the binding pocket and G323E does so to an even greater extent (*p* = 0.008) (Table 2).

**Table 2:** Total Binding Free Energy Values in kJ/mol for Belzutifan in Each of Five Holo-, Holo-G323E, and Holo-p Replicates, with the Mean ± Standard Deviation of the Five Values Shown

	Belzutifan Binding Free Energy (kJ/mol)		
Replicate	Holo	Holo-G323E	Holo-p
1	–132.5 ± 15.1	–75.3 ± 15.3	–86.8 ± 12.8
2	–113.4 ± 10.9	–60.3 ± 17.5	–88.1 ± 11.8
3	–125.6 ± 11.5	–69.2 ± 16.8	–103.5 ± 14.5
4	–114.9 ± 10.7	–70.9 ± 17.2	–129.8 ± 11.7
5	–135.4 ± 12.7	–87.9 ± 13.9	–96.8 ± 15.1
Mean	–124.4 ± 10.0	–72.7 ± 10.1	–101.0 ± 17.5
Δ		51.7	23.4

**Figure 6:**
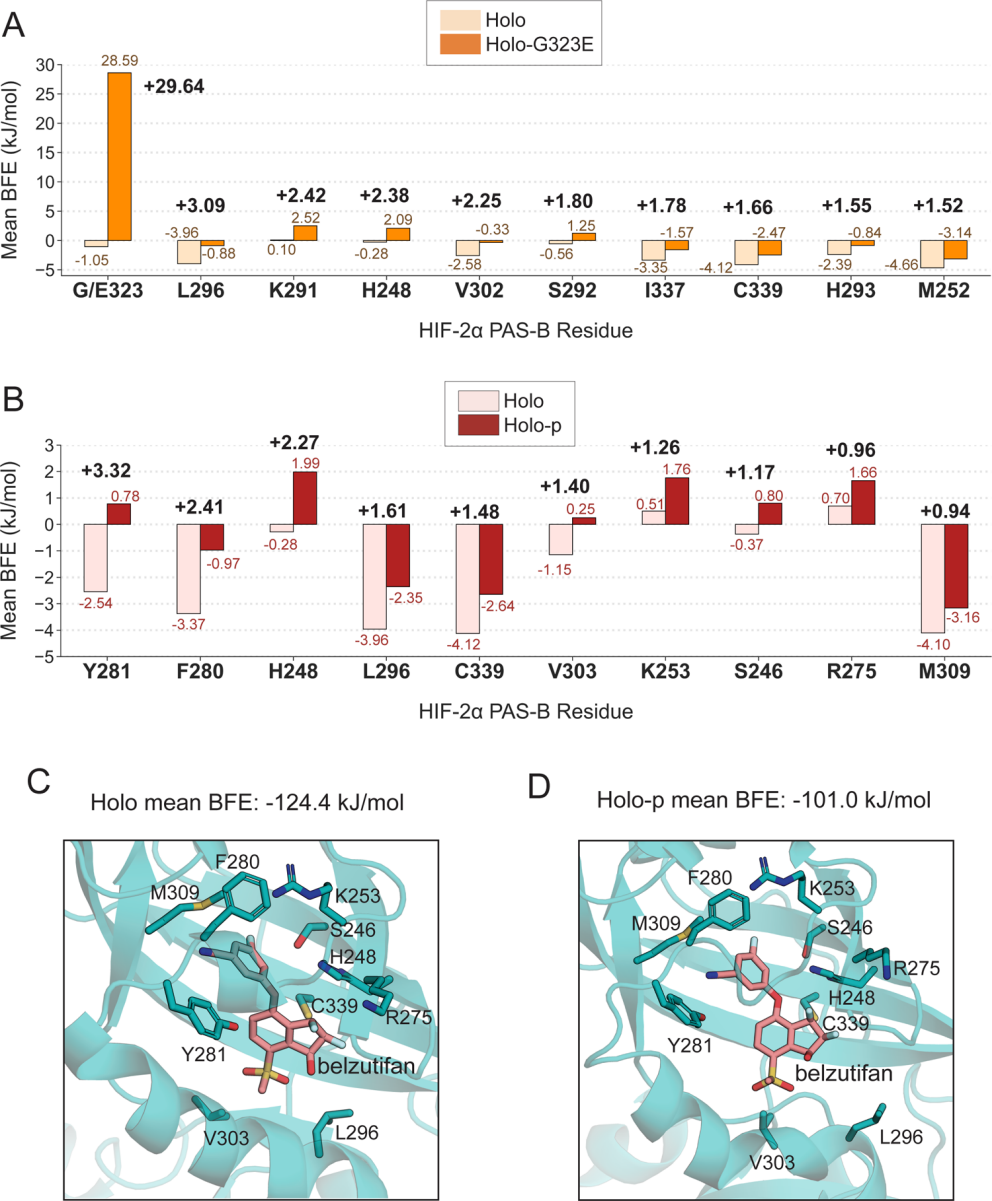
ΔBFE per-residue contributions between holo and holo-p trials. (A) The mean BFE (kJ/mol) values are shown for the top 10 residues that were destabilizing to protein–ligand binding in holo-G323E (orange) relative to holo (light orange). (B) The mean BFE (kJ/mol) values are shown for the top 10 residues that were destabilizing to protein–ligand binding in holo-p (red) relative to holo (pink). (C, D) Belzutifan binding poses in the holo and holo-p complexes are shown in the final frames of holo replicate 1 and holo-p replicate 2, respectively. Stick representations of the top 10 destabilizing residues for holo-p are also shown.

Finally, the per-residue energy decomposition analysis of the total BFE was performed to quantify the destabilizing effects of nearby amino acid residues on protein–belzutifan interactions. In particular, the energy contributions of the ten residues with the highest ΔBFE (mean BFE_holo-G323E/p_ – mean BFE_holo_) further elucidate the allosteric effects of G323E and T324 phosphorylation (Figures 6A,B and S14; Table S13). While the G/E323 residue had an exceptionally large BFE difference of +29.6 kJ/mol, it accounted for only 57.3% of the 51.7 kJ/mol total BFE difference observed in holo-G323E relative to holo, suggesting that rather than solely steric occlusion, more nuanced allosteric effects account for 22.1 kJ/mol (42.7%) of the destabilizing effects of E323 on belzutifan (Figure 6A).

Among the top ten BFE differences for holo-p, which ranged between +3.32 and +0.94 kJ/mol, Y281, F280, and H248 were found to have the highest ΔBFE values at +3.32, +2.41, and +2.27 kJ/mol, respectively (Figure 6B). Furthermore, L296, H248, and C339 were among the top ten for both holo-G323E and holo-p, suggesting that these residues may play a similarly critical role in the destabilizing effects during drug binding for G323E and upon phosphorylation at T324 (Figure 6A–D).

## 4 Discussion

Despite promising advances in HIF-2α inhibition leading to the FDA approval of belzutifan, recent research studies and clinical trials have raised the possibility of acquired resistance through the G323E mutation which introduces a bulky side chain with negative charge facing the interior of the belzutifan binding pocket.^20−22,53^ While it is likely that E323 prevents drug binding by steric hindrance, G323E has been shown to stabilize the heterodimer and possesses additional biochemical properties similar to a phosphorylation.^22^ This raises the possibility of more nuanced allosteric effects during drug binding dynamics and HIF-2α:ARNT complex dissociation that have not been fully explored.

Not surprisingly, phosphorylation also plays a key role in regulating HIF-2 dimerization, localization, and activity. For example, the phosphorylation of HIF-2α at serine 384 or serine 672 increases transcriptional activity by roughly 50%, and phosphorylation at threonine 844 has been shown to increase transcriptional activity by approximately 70%.^54−58^ Growing experimental evidence suggests that phosphorylation occurs at or near the drug-binding site of several protein drug targets, raising a concern about its potential impacts on drug efficacy.^24^ Out of all the known functional phosphosites validated through biochemical experiments, phosphorylation of T324 by PKD1, which putatively disallows HIF-2α from binding Sp1, is uniquely situated within 12 Å of the HIF-2α PAS-B drug binding pocket and shares similarities with the adjacent G323E known to induce drug resistance, making it an attractive phosphosite for analyzing drug binding.^23^ While a Y307 phosphosite also situated within 12 Å of the same binding pocket was detected in a high-throughput screen reported in the dbPTM database, the lack of multiple high-throughput observations, any low-throughput biochemical analyses establishing biological function, and relative acceptance in the literature at the time of writing precluded further study.^59,60^

In this study, we employed MD simulation to investigate and compare the effects of both the G323E mutation and T324 phosphorylation on the belzutifan-bound HIF-2α:ARNT complex. The study reveals pronounced G323E and phosphorylation-induced conformational and energetic changes in the complex as well as previously unreported likely effects of phosphorylation on the interaction between HIF-2α:ARNT and FDA-approved drug belzutifan.

### 4.1 Effects of G323E and pT324 on the HIF-2α:ARNT Dimer Structure

We observed several reproducible dynamic effects of the G323E mutation and T324 phosphorylation on the overall structure of HIF-2α:ARNT complexed with belzutifan. While increased flexibility from G323E was restricted to the Fα helix in HIF-2α PAS-B, a uniform increase in flexibility for multiple structures directly contacting belzutifan was observed for T324 phosphorylation. With the help of a detailed analysis of H-bond contacts of E323/pT324 with surrounding H-bond acceptors, we discovered a novel H-bond contact between G323E and S304 in the HIF-2α PAS-B domain. In holo-p, the increased flexibility of the HIF-2α PAS-B domain is attributed to a dynamic tugging motion between pT324 and N178/R179 located on the HIF-2α PAS-A GH loop, which expands the HIF-2α PAS-B drug-binding pocket and perturbs nearby structured regions involved in HIF-2α:ARNT dimerization (Figure 3). Increased flexibility of structured helices comprising the DNA-binding bHLH domains was also observed for both proteoforms, whereas increased flexibility in the PAS-A domains was largely restricted to the disordered loops.

We also used an ensemble ML approach to analyze the effects of G323E and pT324 on protein interfaces that are known to be critical for stabilizing the HIF-2α:ARNT heterodimer. ML was highly effective in parsing the high-dimensional data from multiple MD replicates using co-residue interactions at each heterodimeric or intramolecular interface and, when combined with MM/PBSA results, revealed whether an important residue participated in more or less favorable interactions between holo-G323E/holo-p and holo replicates. In some cases, agreement between the two methods was largely supported by previously published experimental results.^28^ Several additional residues with an overlapping positive correlation between ML and MM/PBSA were also identified here for the first time and may provide useful targets for future experimental investigation. There were also a handful of cases where MM/PBSA revealed a significant stabilizing effect (≤−2 kJ/mol) for residues which were not marked as important by ML, as in the case of R366, which was highly stabilizing in both holo-G323E and holo-p and found through co-IP to be essential for dimer stability (Table S11). Unlike pT324, which was category 3 and had a large stabilizing effect on the heterodimer, the G/E323 residue was not deemed important by ML yet had a large protein–protein stabilizing effect of −41.1 kJ/mol (Table S5), consistent with experimental reports of G323E stabilizing the HIF-2α:ARNT heterodimer.^22^

### 4.2 Effects of E323 and pT324 on the Belzutifan Binding Pose and Energetics

While TR-FRET analysis by Wu et al. suggested that G323E simply restricts the access of belzutifan analog PT2385 binding to the pocket,^22^ our MM/PBSA data revealed that allosteric effects accounted for roughly 42.7% of the destabilization of belzutifan by G323E, revealing a more nuanced structural and biochemical mechanism by which G323 may prevent belzutifan binding. Interestingly, the same S304 that was found to H-bond to E323 did not H-bond to belzutifan in any of the holo-G323E replicates despite remaining intact in the holo and holo-p replicates, raising the question of whether E323 competes with belzutifan for H-bonding to S304, thereby destabilizing the belzutifan binding pose (Figures 3A,B and S12).

Like G323E, phosphorylation at T324 has a pronounced effect on both the binding pose and binding affinity of belzutifan to HIF-2α:ARNT. Upon phosphorylation, we observed an overall decrease in the number of H-bond donor/acceptor pairs as well as a redistribution of pairing strengths consistent with our finding that pT324 decreases the belzutifan binding affinity, albeit to a lesser extent than G323E (Table 2). Destabilization of belzutifan by pT324, which introduced negative charge and H-bond acceptors facing outward from the HIF-2α PAS-B domain, was explained by an increase in the phosphorylation-induced structural flexibility of HIF-2α PAS-B, localized to residues in not just the Fα helix as in G323E but in the Gβ, Hβ, and Iβ structural elements which all contained residues (L296 in Fα; V303 and M309 in Gβ; C339 in Iβ) found to be less stabilizing of belzutifan after T324 phosphorylation (Figure 6). Notably, decreased flexibility of the Fα and Gβ regions has been previously reported in response to drug binding, further confirming the importance of these structures for HIF-2α:ARNT drug interactions.^52^ Moreover, we also explored the interaction of belzutifan with two distinct neutral protonation states of H293 in holo and holo-p simulations. In both protonation states, pT324 was found to disrupt a key H-bond contact between belzutifan and the H293 residue (Figures S5 and S13). Taken together, these results support the hypothesis that pT324 weakens HIF-2α-belzutifan interactions by uniformly attenuating the structural rigidity of HIF-2α PAS-B and the drug binding pocket, in turn permitting alternate, less stable drug binding positions on the protein.

### 4.3 Caveats and Future Directions

Although G323E is known to likely induce belzutifan resistance, there is a current paucity of clinical data regarding G323E-induced secondary resistance to belzutifan for large cohorts, and the prevalence of the mutation among patients taking belzutifan has not yet been well-established.

While we identified several effects of pT324 on drug binding and protein–protein dynamics, we note that *in vivo* biochemical analysis of T324 phosphorylation from the highly cited work by To et al. was restricted to domain fragments as opposed to the full-length HIF-2α protein.^23^ Though we were unable to find corroborating evidence of T324 phosphorylation in large-scale phosphoproteomic data sets, the relative acceptance of the site in the literature and the present study provide a compelling rationale for further experimental study establishing the presence and stoichiometry of pT324 in humans as well as its effect on drug binding to HIF-2α.

Overall, these findings could inform efforts to design HIF-2 inhibitors that bind more stably to the G323E and/or T324-phosphorylated target and highlight the utility of molecular dynamics as a cost-effective approach to prioritizing phosphosites for *in vitro* drug binding experiments.

## Data Availability

The codes used to run the ML model are available at https://github.gatech.edu/mtorres35/HIF-2_T324. Training data and other codes are available by request.

## Supporting Information Available

List of additional molecular dynamics, binding free energy, and machine learning analyses along with molecular dynamics movies (PDF)
Movie showing tugging motion of N178 and R179 on phosphorylated T324. The last 250 ns of 5 holo-p replicates were concatenated to form the 1.25 μs trajectory shown. Only the HIF-2α protomer is shown while the ARNT protomer is hidden. (MPG)
Movie showing lack of tugging motion of N178 and R179 on unphosphorylated T324. The last 250 ns of 5 holo replicates were concatenated to form the 1.25 μs trajectory shown. Only the HIF-2α protomer is shown while the ARNT protomer is hidden. (MPG)

## Supplementary Material

ao4c03777_si_001.pdf

ao4c03777_si_002.mpg

ao4c03777_si_003.mpg

## Abbreviations

ARNT: aryl hydrocarbon receptor nuclear translocator
ccRCC: clear cell renal cell carcinoma
HIF: hypoxia inducible factor
HRE: hypoxia response element
VHL: von Hippel-Lindau
BFE: binding free energy
