# Supplementary material for: Molecular Dynamics Reveals Altered Interactions between Belzutifan and HIF-2 with Natural Variant G323E or Proximal Phosphorylation at T324
Source: ACS Omega. 2024 Aug 26;9(36):37843–55. doi: 10.1021/acsomega.4c03777 (PMC11391435; doi:10.1021/acsomega.4c03777)
Supplement: Supplementary file 1 — ao4c03777_si_001.pdf [file ao4c03777_si_001.pdf]

Supporting Information

Molecular dynamics reveals altered interactions between belzutifan and HIF-2 with natural variant G323E or proximal phosphorylation at T324

Vishva Natarajan[1], Vardhan Satalkar[1,*], James C. Gumbart[2], and Matthew Torres[1,*]

[1]School of Biological Sciences, Georgia Institute of Technology, Atlanta, GA 30332

[2]School of Physics, Georgia Institute of Technology, Atlanta, GA 30332

[*]Corresponding Authors: Vardhan Satalkar (vsatalkar3@gatech.edu) and Matthew P. Torres (mtorres35@gatech.edu)

Additional Supplementary data and codes are available at GitHub link: https://github.gatech.edu/mtorres35/HIF-2_T324

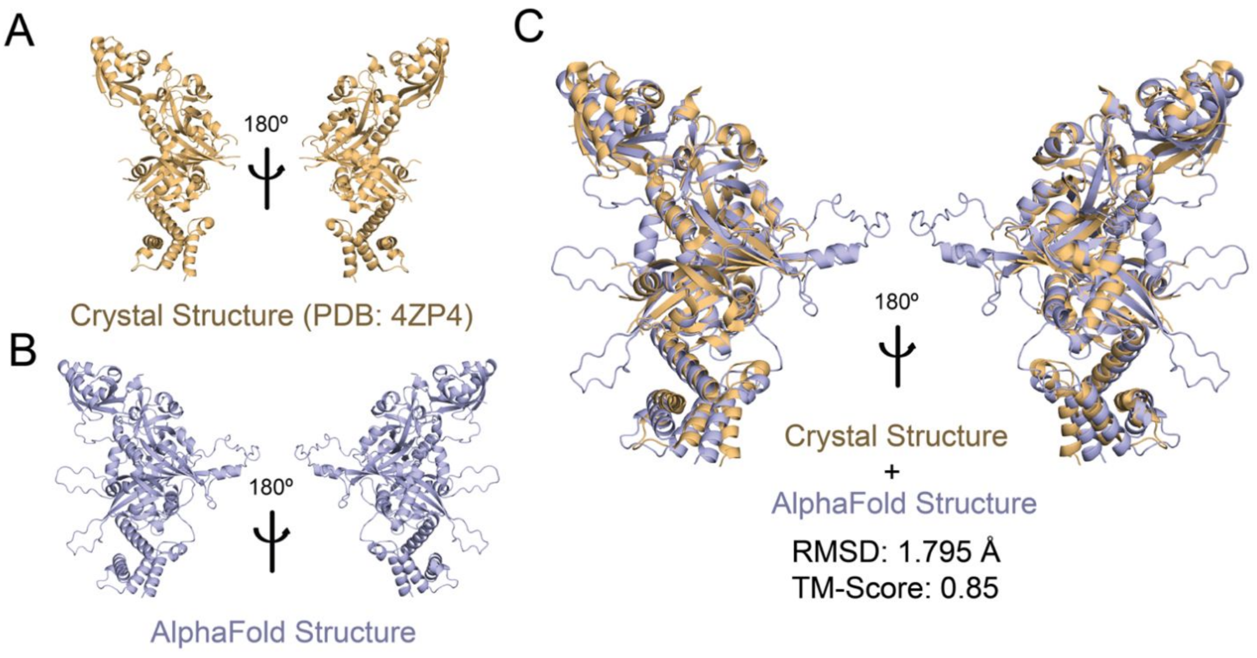

D

HIF-2α

```
26  RRSKETEVFYELAHELPLPHSVSSHLDKASIMRLAISFLRTHKLLSSVCS----------  85
    RRSKETEVFYELAHELPLPHSVSSHLDKASIMRLAISFLRTHKLLSSVCSENESEAEADQ

86  -MDNLYLKALEGFIAVVTQDGDMIFLSENISKFMGLTQVELTGHSIFDFTHPCDHEEIRE  145
    QMDNLYLKALEGFIAVVTQDGDMIFLSENISKFMGLTQVELTGHSIFDFTHPCDHEEIRE

146 NLTL------------VSTERDFFMRMKCTVTNRGRTVNLKSATWKVLHCTGQVRV----  205
    NLTLKNGSGFGKKSKDVSTERDFFMRMKCTVTNRGRTVNLKSATWKVLHCTGQVRVYNNC

206 -------------LSCLIIMCEPIQHPSHMDIPLDSKTFLSRHSMDMKFTYCDDRILELI  265
    PPHSSLCGSKEPLLSCLIIMCEPIQHPSHMDIPLDSKTFLSRHSMDMKFTYCDDRILELI

266 GYHPEELLGRSAYEFYHALDSENMTKSHQNLCTKGQVVSGQYRMLAKHGGYVWLETQGTV  325
    GYHPEELLGRSAYEFYHALDSENMTKSHQNLCTKGQVVSGQYRMLAKHGGYVWLETQGTV

326 IYNPRNLQPQCIMCVNYVLSEIEKNDVVFSMDQTE 360
    IYNPRNLQPQCIMCVNYVLSEIEKNDVVFSMDQTE
```

ARNT

```
98  ERRRRNKMTAYITELSDMVPTCSALARKPDKLTILRMAVSHMKSL---------------  157
    ERRRRNKMTAYITELSDMVPTCSALARKPDKLTILRMAVSHMKSLRGTGNTSTDGSYKPS

158 -LTDQELKHLILEAADGFLFIVSCETGRVVYVSDSVTPVLNQPQSEWFGSTLYDQVHPDD  217
    FLTDQELKHLILEAADGFLFIVSCETGRVVYVSDSVTPVLNQPQSEWFGSTLYDQVHPDD

218 VDKLREQLSTS------------------------------SRRSFICRMRCGT------  277
    VDKLREQLSTSENALTGRVLDLKTGTVKKEGQQSSMRMCMGSRRSFICRMRCGTSSVDPV

278 -----------------------PHFVVVHCTGYIKAW------------------KFCL  337
    SMNRLSFLRNRCRNGLGSVKEGEPHFVVVHCTGYIKAWPPAGVSLPDDDPEAGQGSKFCL

338 VAIGRLQVT-------------PTEFISRHNIEGIFTFVDHRCVATVGYQPQELLGKNIV  397
    VAIGRLQVTSSPNCTDMSNICQPTEFISRHNIEGIFTFVDHRCVATVGYQPQELLGKNIV

398 EFCHPEDQQLLRDSFQQVVKLKGQVLSVMFRFRSKTREWLWMRTSSFTFQNPYSDEIEYI  457
    EFCHPEDQQLLRDSFQQVVKLKGQVLSVMFRFRSKTREWLWMRTSSFTFQNPYSDEIEYI

458 ICTNTNV 464
    ICTNTNV
```

Figure S1. Initial protein structure of the HIF-2α:ARNT complex. (A) Front and back view of the 4ZP4 apo crystal structure, colored tan. (B) Front and back view of the AlphaFold-predicted structure, colored light blue. (C) PyMol-generated overlay of the AlphaFold structure (light blue) onto the crystal structure (tan). The RMSD and TM-Score between the AlphaFold model and the crystal structure were computed to be 1.795 Å and 0.85, respectively. (D) Pairwise alignment between 4ZP4

and AlphaFold sequence is shown, and residues missing in 4ZP4 but present in AlphaFold structure are highlighted in yellow.

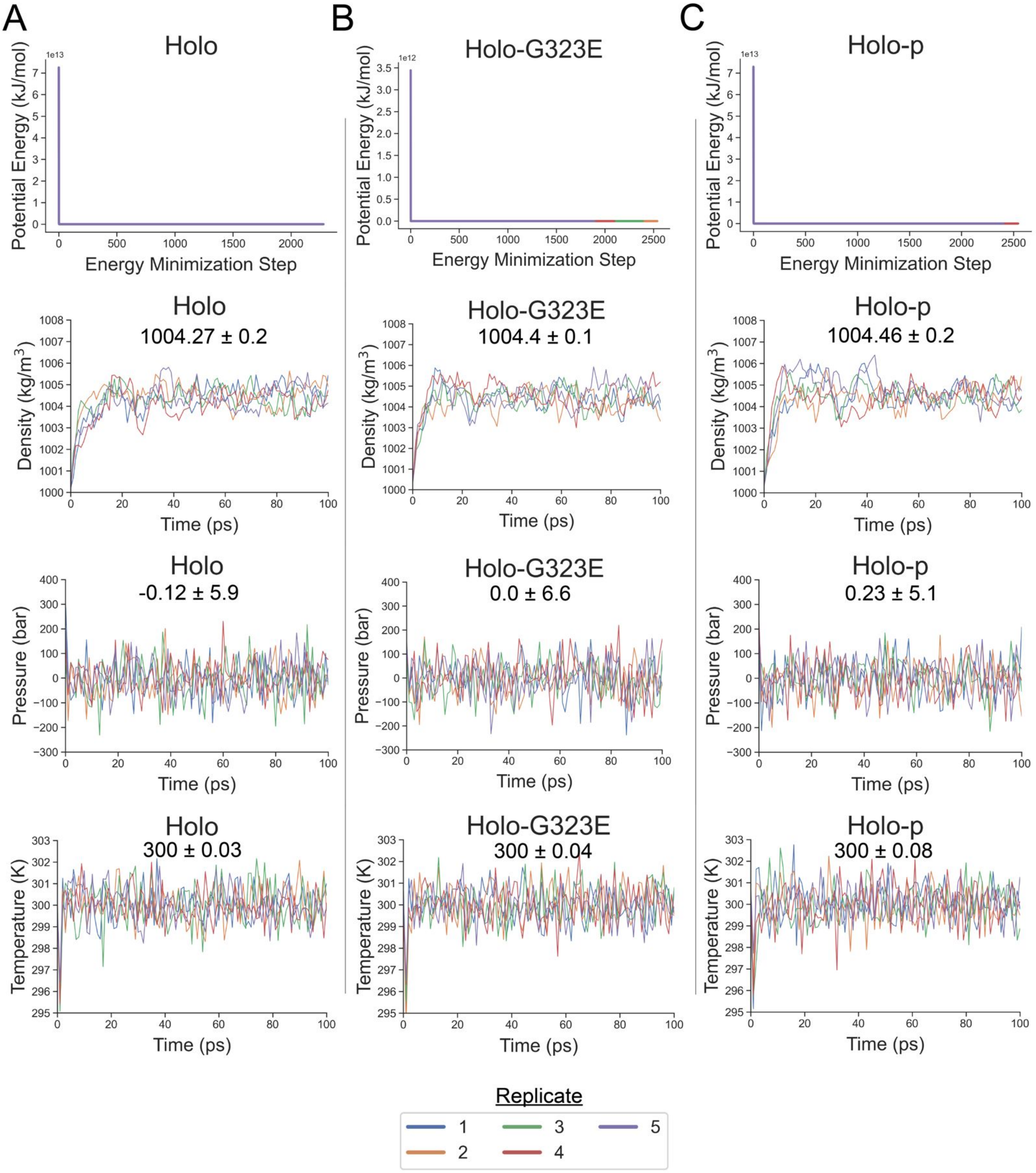

Figure S2. Analysis of the MD simulation parameters, namely potential energy values (kJ/mol), density (kg/m3), total pressure (bar), and temperature (K) of the system are plotted for the five MD replicates of (A) holo, (B) holo-G323E, and (C) holo-p complexes against timestep (ps). The convergence of each parameter was determined using average and standard deviation values indicated in the density, pressure, and temperature subplots.

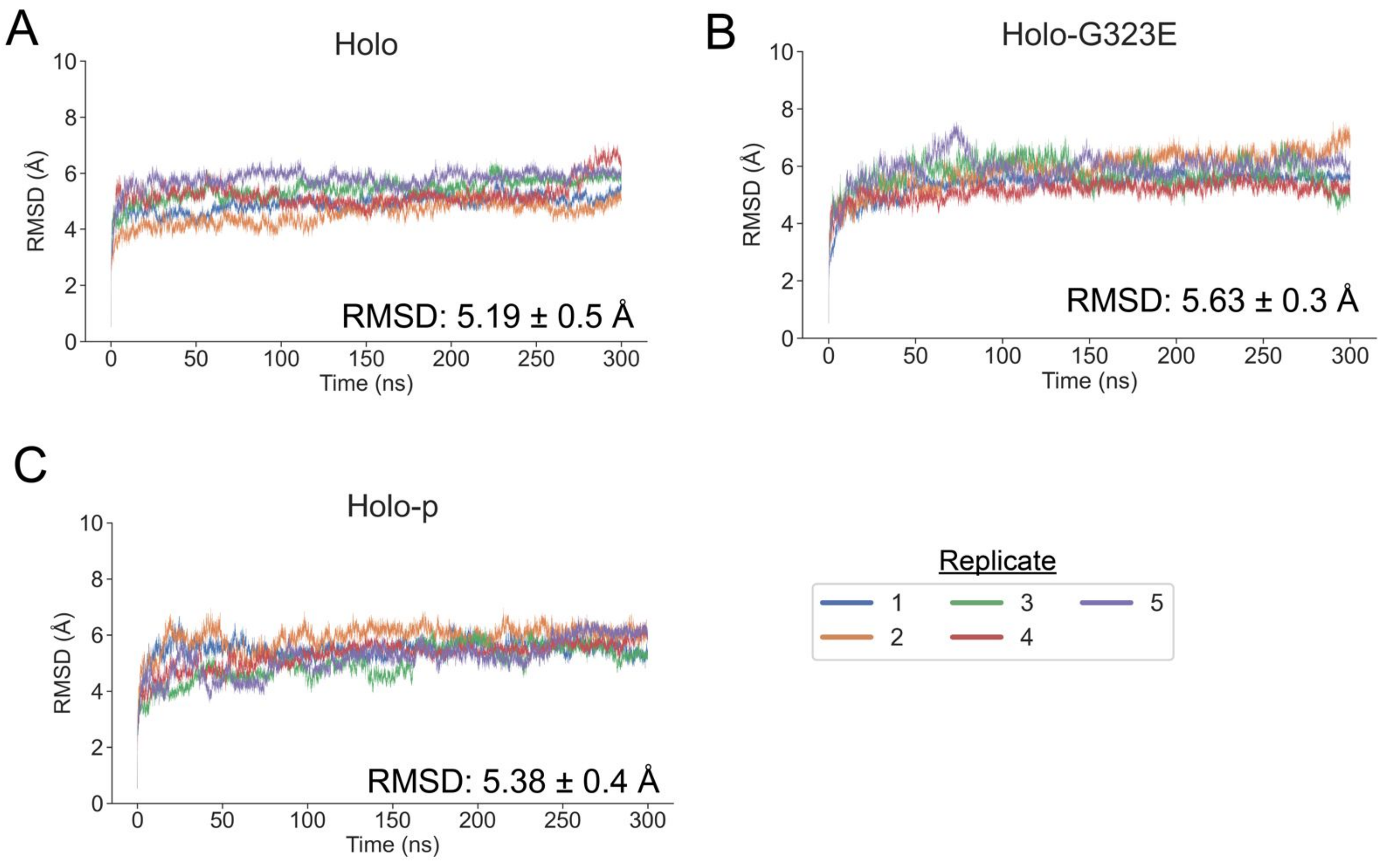

Figure S3. Backbone RMSD (Å) for (A) Holo (B) Holo-G323E and (C) Holo-p protein simulation replicates are shown with each replicate color-coded in the legend. Mean ± standard deviation of the mean RMSD is shown. RMSD values of all replicates converge after 50 ns.

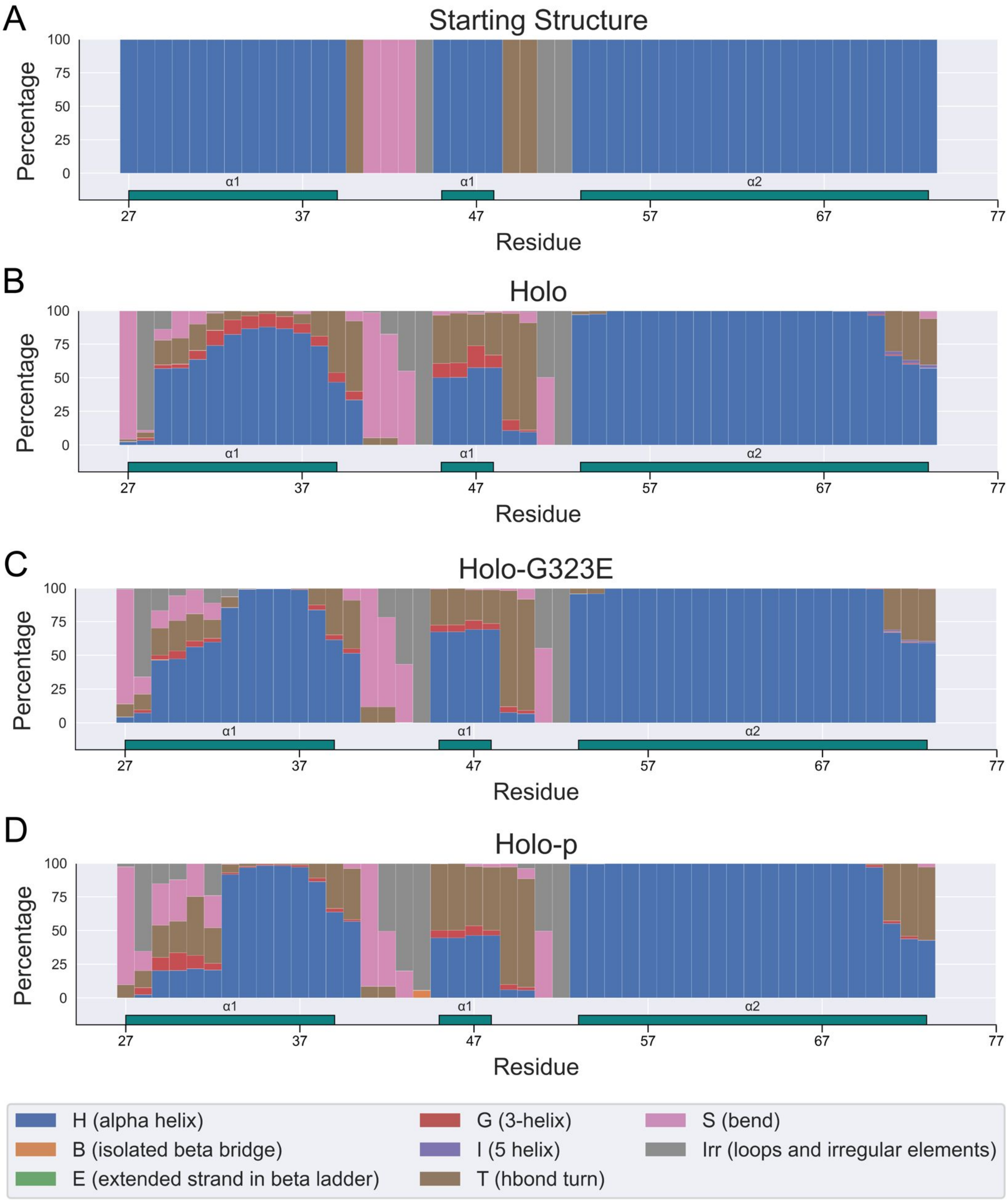

Figure S4. Secondary structure analysis of HIF-2α bHLH domain. The frequency of the secondary structure assignments is shown for residues in the HIF-2α bHLH domain in (A) AlphaFold2 initial structure; (B) the holo, (C) holo-G323E, and (D) holo-p simulations.

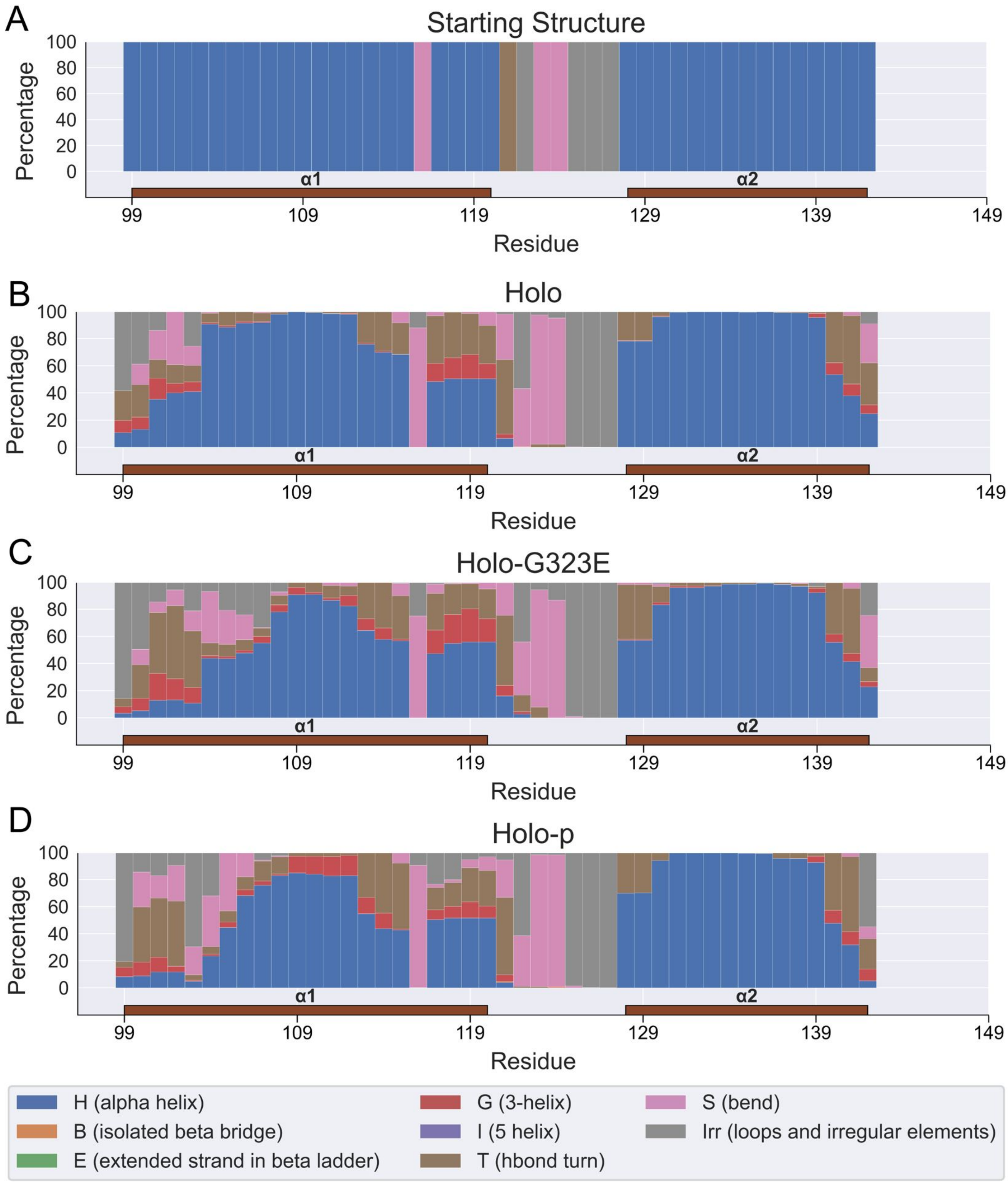

Figure S5. Secondary structure analysis of ARNT bHLH domain. The frequency of the secondary structure assignments is shown for residues in the ARNT bHLH domain in (A) AlphaFold2 initial structure; (B) the holo, (C) holo-G323E, and (D) holo-p simulations.

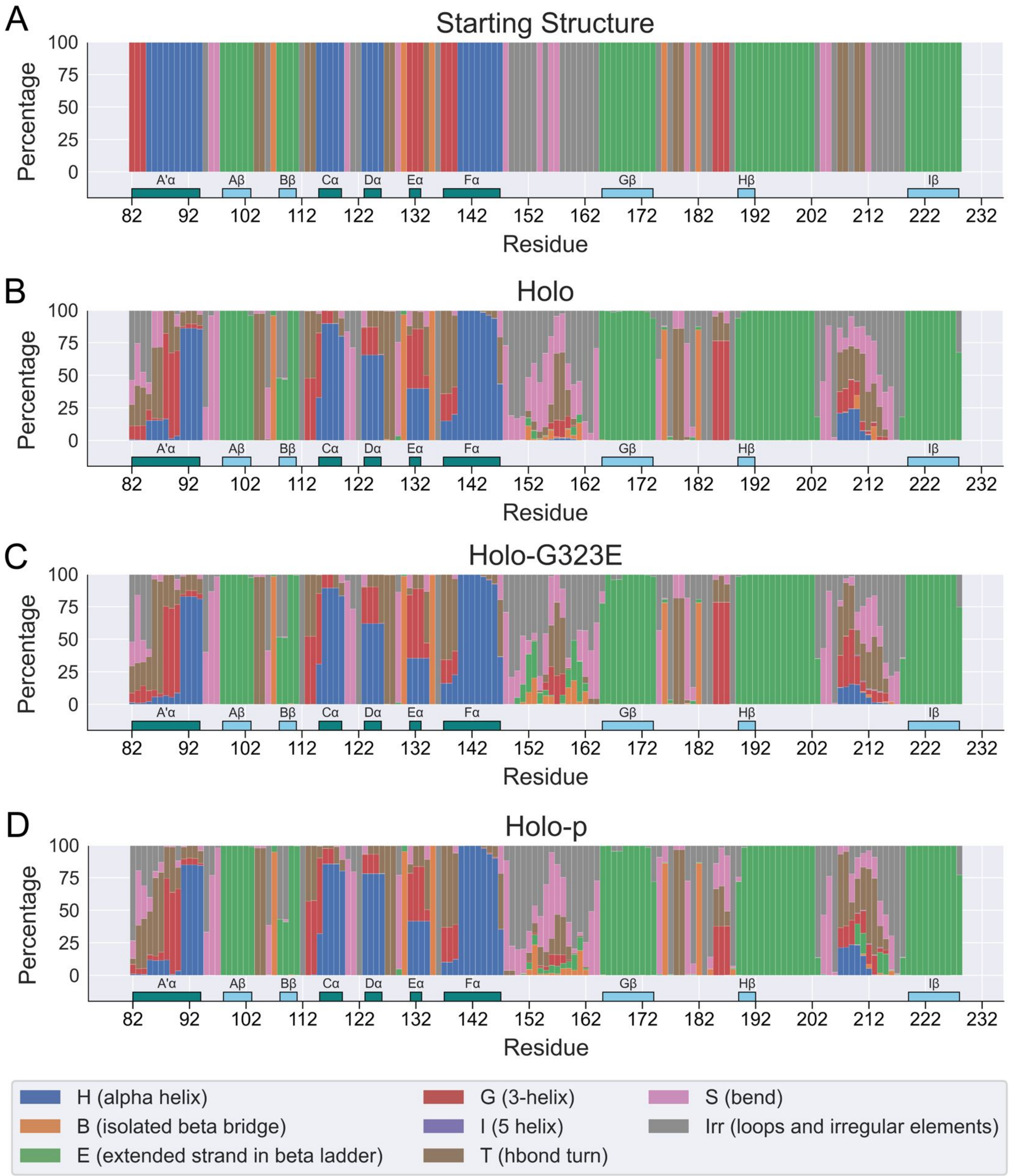

Figure S6. Secondary structure analysis of HIF-2α PAS-A domain. The frequency of the secondary structure assignments is shown for residues in the HIF-2α PAS-A domain in (A) AlphaFold2 initial structure; (B) the holo, (C) holo-G323E, and (D) holo-p simulations.

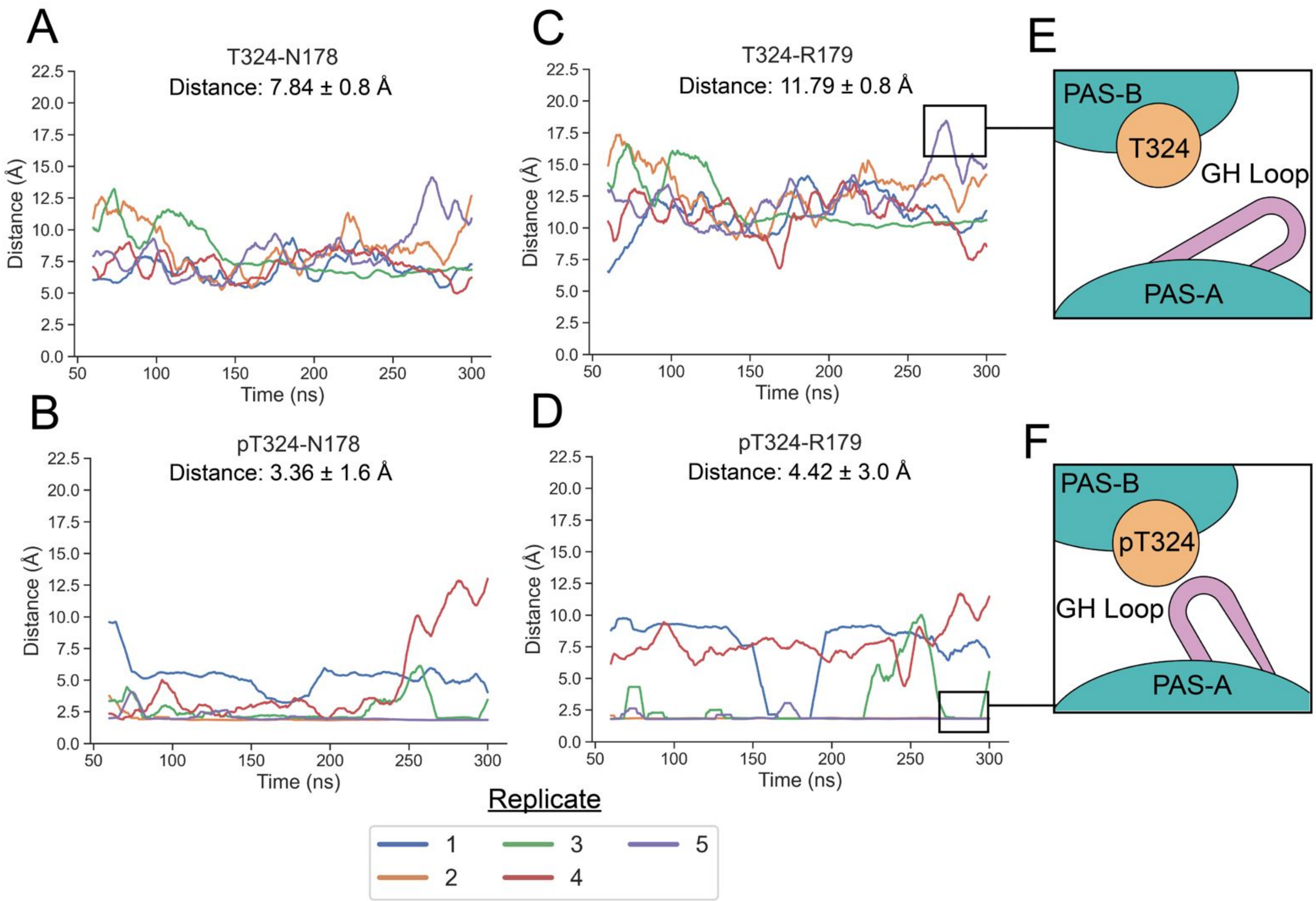

Figure S7. Key H-bond interactions between either T324 or pT324 with GH loop residues N178 and R179. 1000-point moving average computed using side-chain distance to N178 for (A) T324 and (B) pT324 as well as to R179 for (C) T324 and (D) pT324. Mean ± standard deviation is shown for each distance plot. Schematic showing position of GH loop (E) away from HIF-2α PAS-B in holo compared to (F) towards HIF-2α PAS-B in holo-p. All distances calculated using last 250 ns of each trajectory.

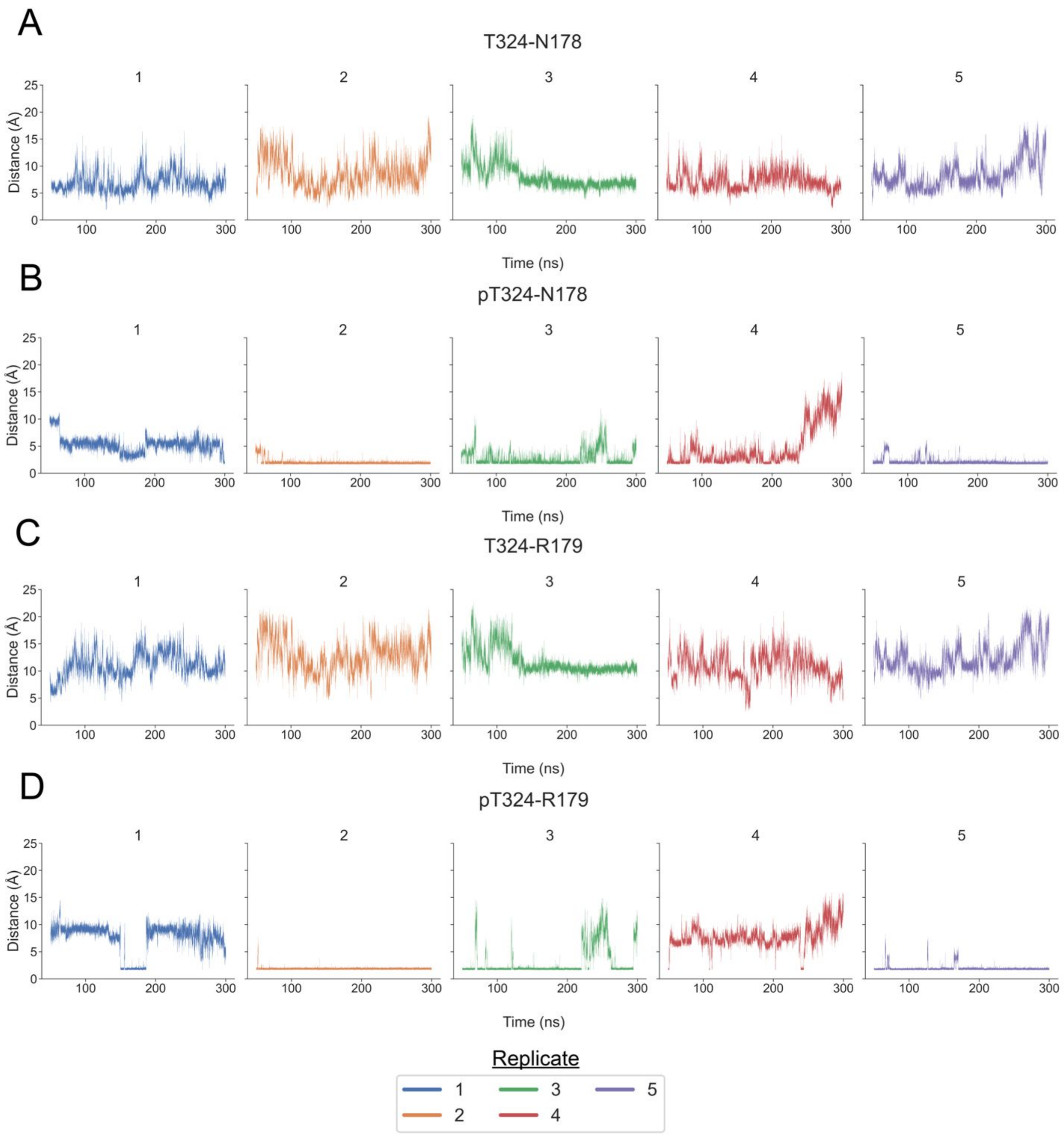

Figure S8. The residue-residue distances between H-bond acceptor T324/pT324 and H-bond donors (A-B) N178 residue; (C-D) R179 residues are shown for each of 5 holo and holo-p replicates. MD replicate number are color-coded in legend.

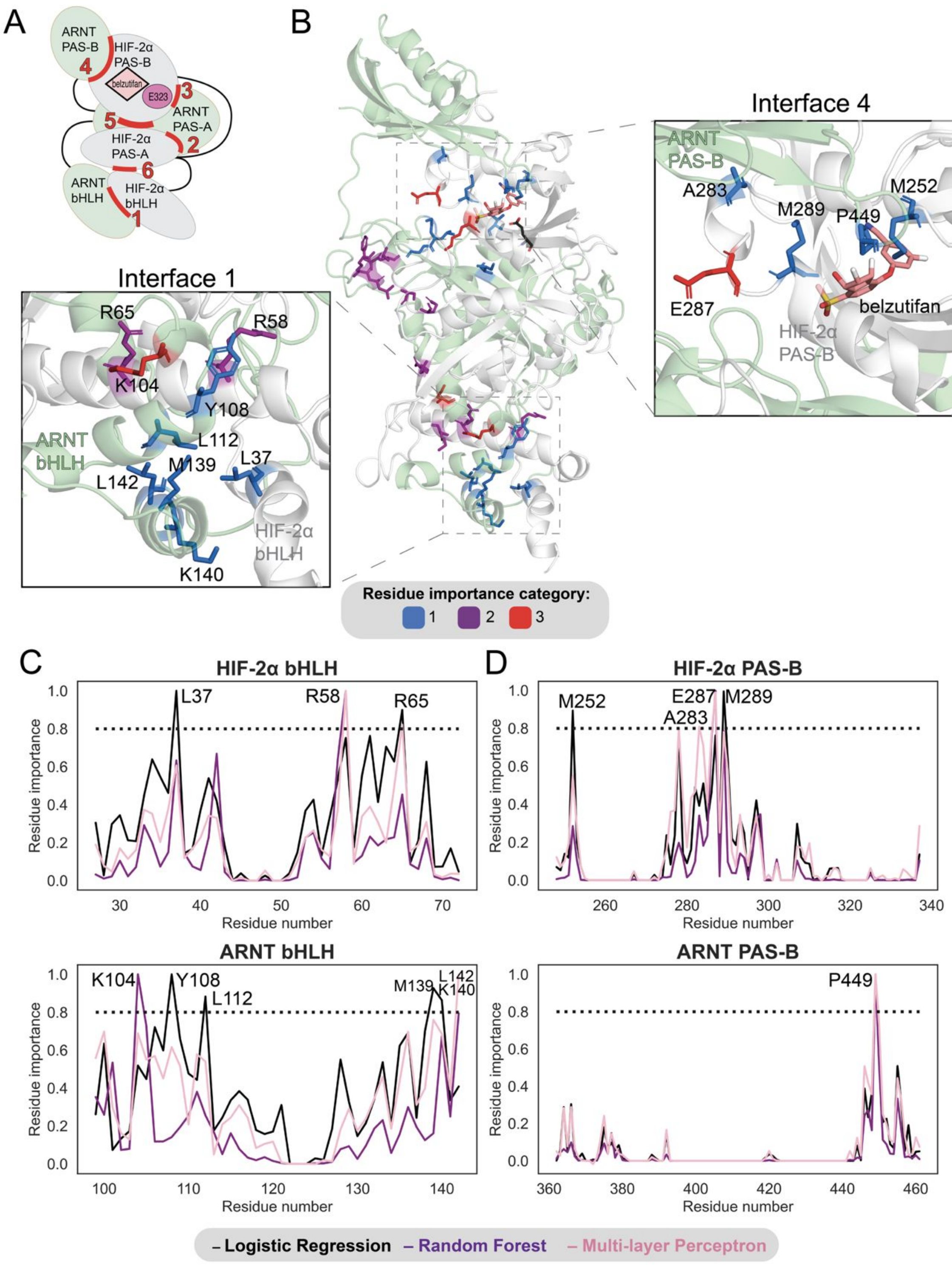

Figure S9. ML results combined with ΔG_{Total} and ΔRMSF for holo-G323E vs holo. (A) Schematic of HIF-2α:ARNT domain architecture with six interfaces labelled. (B) Residue importance categories labelled on representative structure from holo-G323E replicate. Category 1 residues had importance >0.8 in one of the three ML models (Logistic Regression (LR), Random Forest (RF), and Multi-Layer Perceptron (MLP)) and are shown in blue color. Category 2 residues also had either absolute ΔG_{Total} ≥2 kJ/mol or ΔRMSF ≥1 Å (≥3 Å for disordered regions) and are shown in purple color. Category 3 residues met all three criteria and are shown in red color. Per-residue importance scores from each ML model are shown for (C) interchain interface 1 (HIF-2α bHLH: ARNT bHLH) and (D) interchain interface 4 (HIF-2α PAS-B: ARNT PAS-B). Y-axis is residue importance score while x-axis is residue number. Black line is LR, purple line is RF, and pink line is MLP. Dotted line at importance cutoff of 0.8.

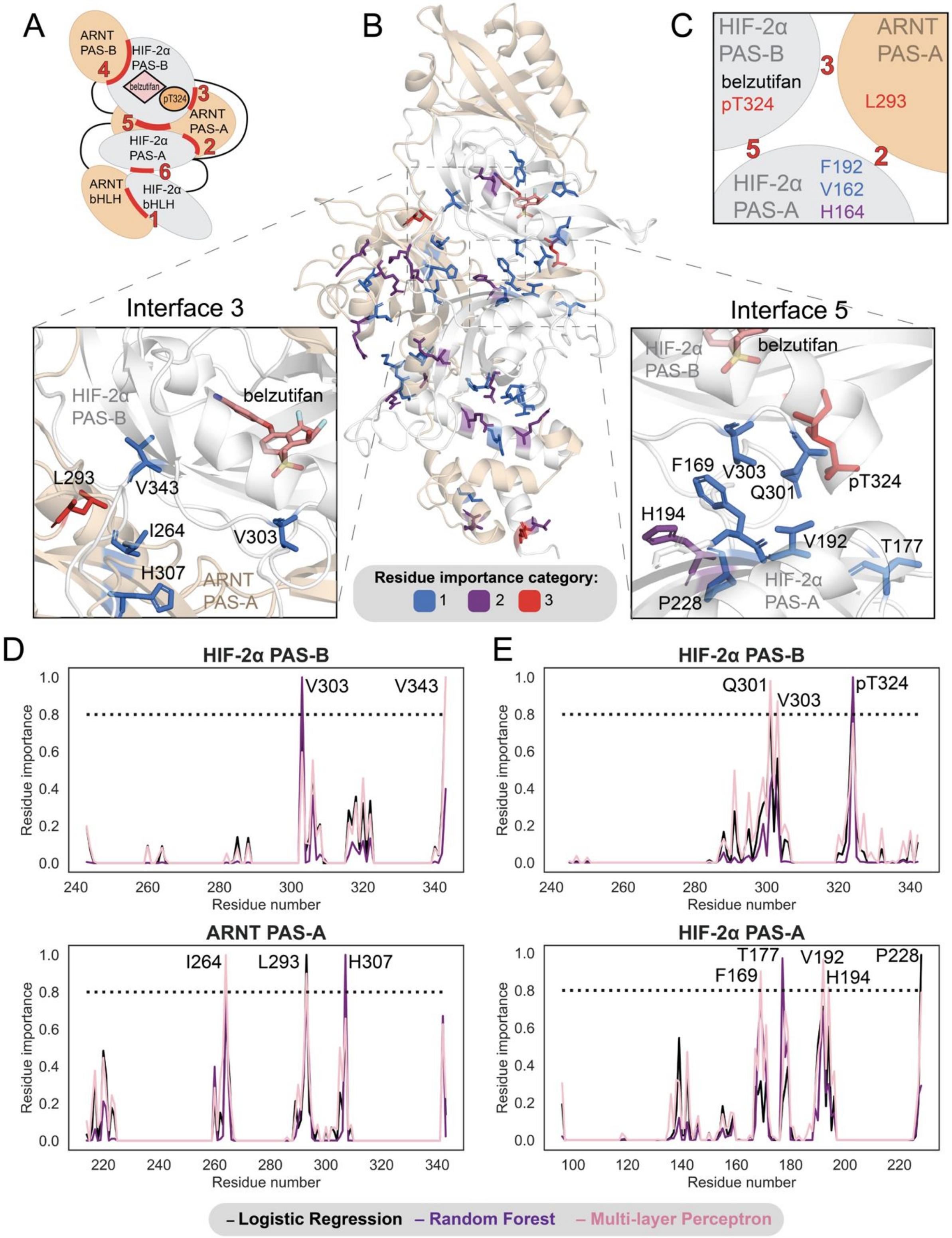

Figure S10. ML results combined with ΔG_{Total} and ΔRMSF for holo-p vs holo. (A) Schematic of HIF-2α:ARNT domain architecture with six interfaces labelled. (B) Residue importance categories labelled on representative structure from holo-p replicate. Category 1 residues had importance >0.8 in one of the three ML models (Logistic Regression (LR), Random Forest (RF), and Multi-Layer Perceptron (MLP)) and are shown in blue color. Category 2 residues had either absolute ΔG_{Total} ≥2 kJ/mol or ΔRMSF ≥1 Å (≥3 Å for disordered regions) and are shown in purple color. Category 3 residues met all three criteria and are shown in red color. (C) 3D orientation of key interfaces identified by co-immunoprecipitation[28] and modulated by pT324. Per-residue importance scores from each ML model are shown for (D) interchain interface 4 (HIF-2α PAS-B: ARNT PAS-B) and (E) intrachain interface 5 (HIF-2α PAS-B:PAS-A). Y-axis is residue importance score while x-axis is residue number. Black line is LR, purple line is RF, and pink line is MLP. Dotted line at importance cutoff of 0.8.

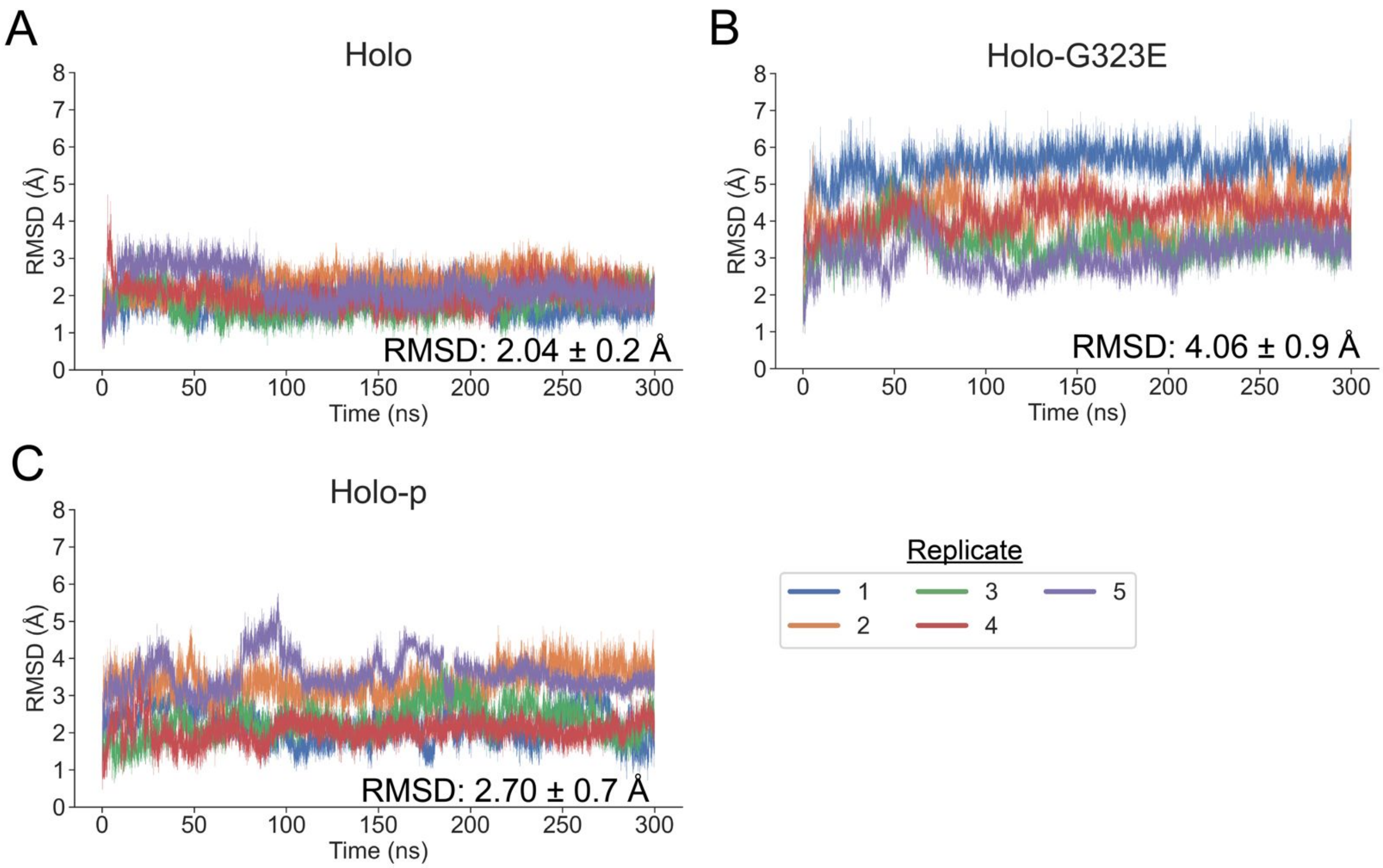

Figure S11. Ligand flexibility in the binding pocket. Belzutifan RMSD (Å) is shown after fitting to protein backbone for (A) Holo (B) Holo-G323E and (C) Holo-p as well as the mean ± standard deviation of mean RMSD. MD replicate number are color-coded in legend.

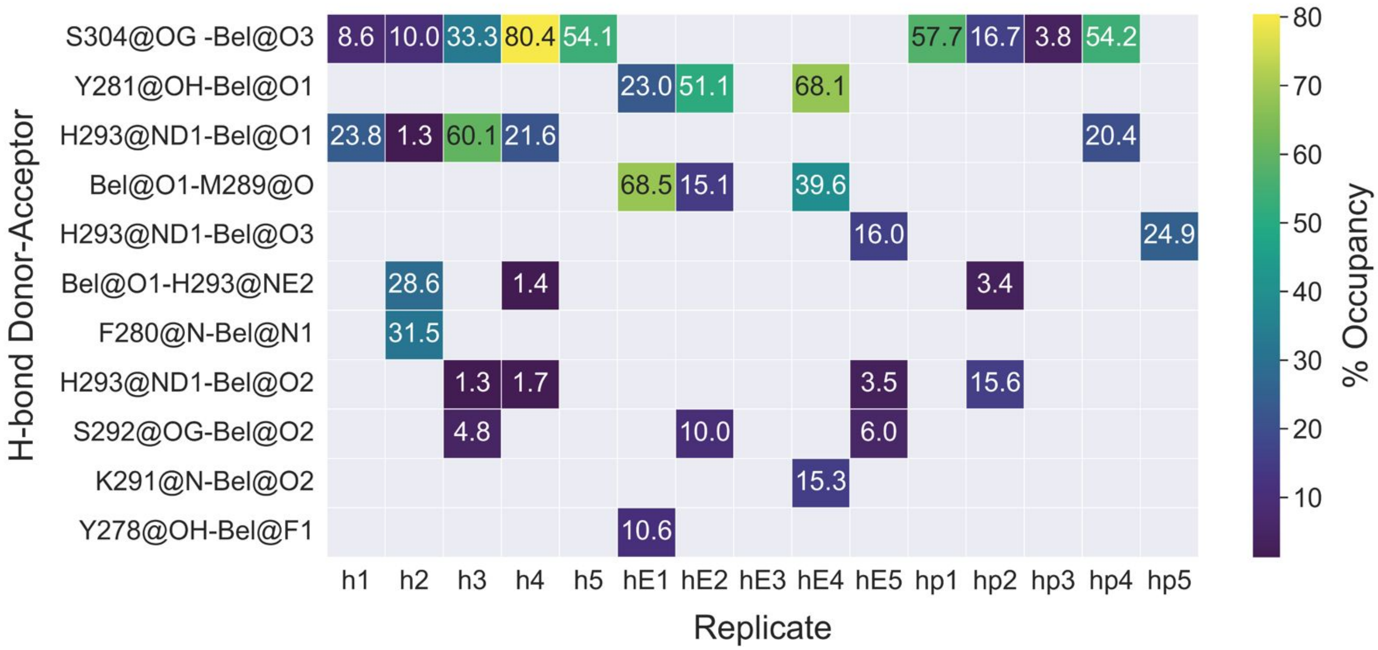

Figure S12. Heatmap showing % H-bond occupancies for all donor-accepter pairs with at least 10% occupancy in at least one holo (h1-h5), holo-G323E (hE1-hE5), or holo-p (hp1-hp5) replicate.

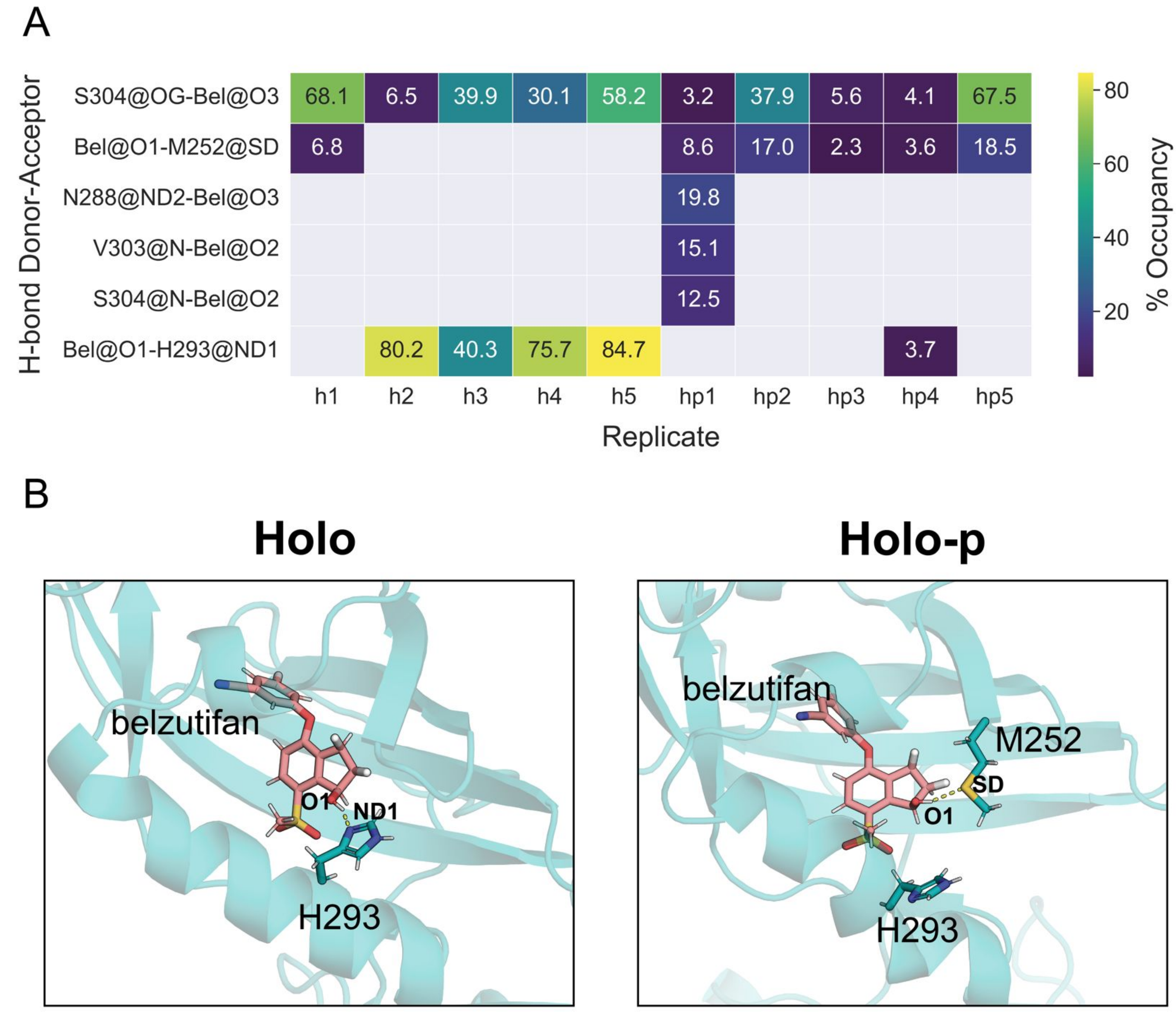

Figure S13. H-bond occupancies for the belzutifan ligand in the HIF-2α PAS-B binding pocket with the H293 set to HIE using GROMACS pdb2gmx tool. After setting H293 of HIF-2α to HIE, 5 additional replicates each of holo and holo-p 300 ns simulations were performed for a total of 10 simulations and 3 μs total simulation time. (A) Heatmap showing H-bond occupancies for all donor-accepter pairs with at least 10% occupancy in at least one holo or holo-p replicate. (B) Representative structures from holo and holo-p replicate 5 showing belzutifan O1 atom donating H-bond to H293 ND1 atom in holo replicates, whereas in holo-p replicates the belzutifan O1 atom donates H-bond to SD atom of M252.

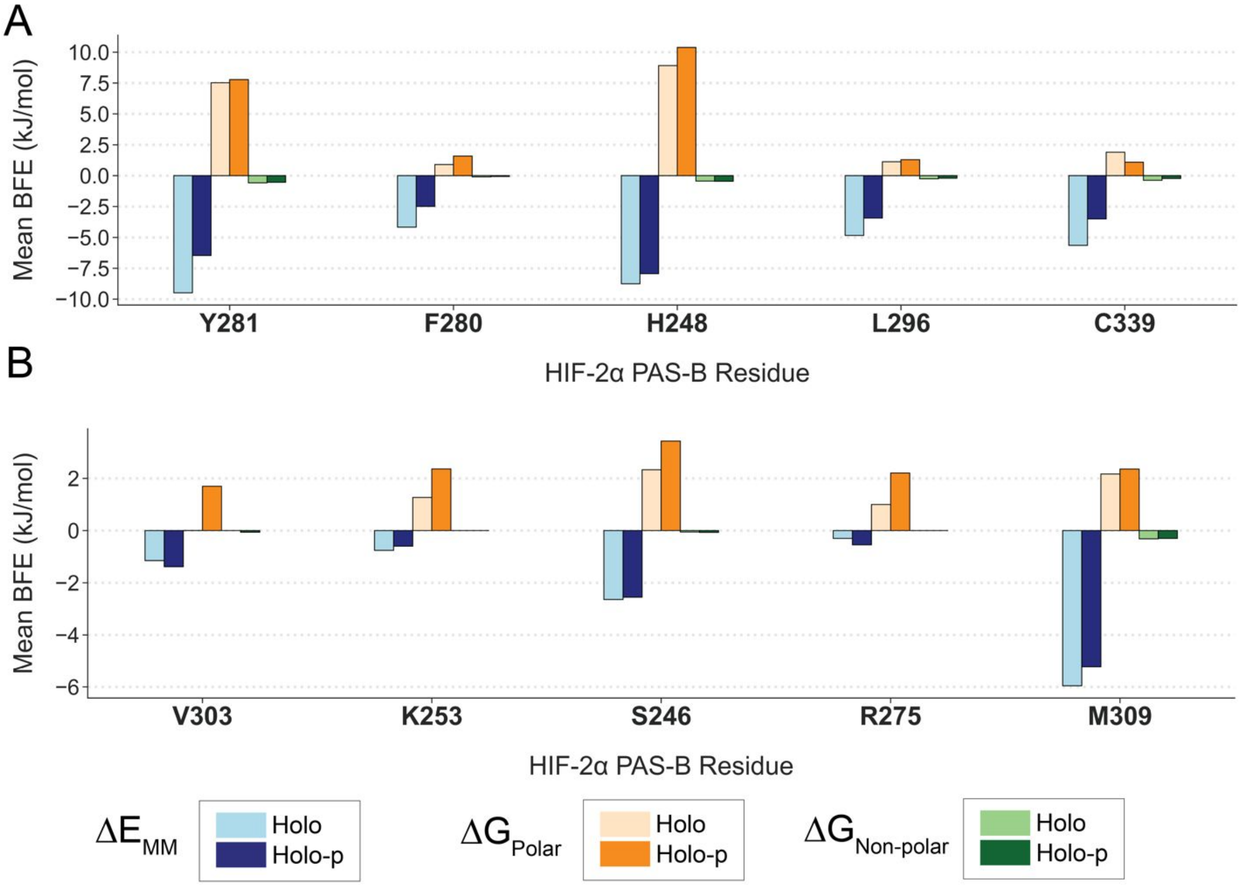

C

		ΔE_{MM} (kJ/mol)			ΔG_{Polar} (kJ/mol)			$\Delta G_{Non\text{-}polar}$ (kJ/mol)		
	Residue	Holo	Holo-p	Δ	Holo	Holo-p	Δ	Holo	Holo-p	Δ
1	Y281	-9.483	-6.467	3.016	7.521	7.781	0.259	-0.583	-0.536	0.047
2	F280	-4.174	-2.491	1.683	0.899	1.589	0.690	-0.100	-0.068	0.032
3	H248	-8.757	-7.937	0.820	8.911	10.383	1.472	-0.436	-0.456	-0.020
4	L296	-4.840	-3.436	1.403	1.125	1.296	0.170	-0.251	-0.213	0.038
5	C339	-5.640	-3.503	2.136	1.897	1.089	-0.808	-0.382	-0.230	0.152
6	V303	-1.150	-1.385	-0.235	0.005	1.698	1.693	-0.001	-0.063	-0.063
7	K253	-0.759	-0.598	0.162	1.266	2.360	1.094	0.000	0.000	0.000
8	S246	-2.638	-2.552	0.087	2.326	3.427	1.101	-0.056	-0.072	-0.016
9	R275	-0.300	-0.547	-0.247	0.996	2.205	1.209	0.000	0.000	0.000
10	M309	-5.955	-5.217	0.739	2.167	2.355	0.189	-0.316	-0.300	0.016

Figure S14. Protein-ligand Binding Free Energy (BFE) components, namely ΔE_{MM} (blue), ΔG_{Polar} (orange), and $\Delta G_{Non\text{-}polar}$ (green), are shown for both holo and holo-p. Mean BFE on y-axis and residue name on x-axis. (A) First five of top 10 residues with the highest ΔG_{Total} and the (B) last five of top 10 residues with the highest ΔG_{Total} are shown in the bar plot. In each bar plot, lighter color is holo while darker color is holo-p. (C) Tabulated values for protein-ligand BFE components ΔE_{MM} , ΔG_{Polar} , and $\Delta G_{Non\text{-}polar}$ for the top 10 residues with the highest ΔG_{Total}. Values are shown for both holo and holo-p, and difference (Δ) was calculated as holo-p minus holo.

Table S1. Ordered region residues in each protomer are listed with absolute difference in Cα-RMSF greater than or equal to 1 Å. RMSF values were calculated using last 250 ns of 5 holo, holo-p, or holo-G323E trials concatenated together.

				RMSF (Å)		
**Holo vs Holo-G323E**						
Protomer	Domain	Secondary Structure	Residue	Holo	Holo-G323E	Difference
HIF-2α	bHLH	α1	S28	4.636	5.911	1.275
	PAS-A	A'α	E82	5.422	6.495	1.073
		A'α	A83	4.943	6.259	1.316
		A'α	D84	3.382	4.531	1.149
	PAS-B	Fα	E287	1.533	2.705	1.172
		Fα	T290	1.530	3.025	1.495
		Fα	K291	1.633	3.552	1.919
		Fα	S292	1.395	3.081	1.686
		Fα	H293	1.372	2.791	1.419
		Fα	Q294	1.376	2.53	1.154
		Fα	N295	1.311	2.543	1.232
		Fα	T298	1.414	2.436	1.022
ARNT	bHLH	α1	R99	10.386	6.736	-3.65
		α1	R100	8.007	5.878	-2.129
		α1	R101	7.614	5.768	-1.846
		α1	K104	4.342	5.642	1.3
		α1	M105	3.712	5.203	1.491
		α2	K128	2.838	4.117	1.279
		α2	L129	2.667	3.718	1.051

Table S1. Ordered region residues in each protomer are listed with absolute difference in Cα-RMSF greater than or equal to 1 Å. RMSF values were calculated using last 250 ns of 5 holo, holo-p, or holo-G323E trials concatenated together.

				RMSF (Å)		
**Holo vs Holo-p**						
Protomer	Domain	Secondary Structure	Residue	Holo	Holo-p	Difference
HIF-2α	bHLH	α1	R27	6.123	8.384	2.261
			S28	4.636	7.249	2.613
			K29	4.331	6.34	2.009
			E30	3.533	6.049	2.516
			T31	3.486	4.908	1.422
			H45	3.014	4.499	1.485
			S46	3.373	4.426	1.053
	PAS-A	A'α	E82	5.422	4.393	-1.029
			D84	3.382	4.74	1.358
			Q85	2.153	3.335	1.182
	PAS-B	Fα	T290	1.53	2.878	1.348
			K291	1.633	2.718	1.085
			H293	1.372	2.449	1.077
			Q294	1.376	2.733	1.357
			C297	1.445	3.142	1.697
			T298	1.414	3.096	1.682
			K299	1.269	2.347	1.078
		Gβ	V302	1.146	2.294	1.148
		Hβ	T324	1.242	2.297	1.055
			V325	1.409	2.504	1.095
			I326	1.599	2.698	1.099
			Y327	1.855	3.032	1.177
		Iβ	P334	1.838	2.932	1.094
ARNT	bHLH	α1	R99	10.386	5.437	-4.949
			R100	8.007	4.542	-3.465
			R101	7.614	4.564	-3.05
			R102	6.659	4.336	-2.323
			N103	5.053	3.555	-1.498
			S113	2.027	3.096	1.069
			P117	2.479	3.628	1.149
			T118	2.692	4.176	1.484
			C119	2.64	4.667	2.027
			S120	3.043	5.901	2.858
		α2	M134	1.993	3.003	1.01
			S137	2.049	3.093	1.044
			K140	2.102	3.224	1.122
			S141	2.22	3.362	1.142

Table S2. Non-terminal disordered region residues in each protomer are listed with absolute difference in Cα-RMSF greater than or equal to 3 Å. RMSF values were calculated using last 250 ns of 5 holo, holo-p, or holo-G323E trials concatenated together.

				RMSF (Å)		
**Holo vs Holo-G323E**						
Protomer	Domain	Structural Element	Residue	Holo	Holo-G323E	Difference
HIF-2α	PAS-A	FG Loop	F155	8.710	12.288	3.578
			G156	9.120	14.134	5.014
			K157	8.948	13.976	5.028
			K158	8.299	12.703	4.404
			S159	6.411	10.254	3.843
			K160	4.754	8.083	3.329
			D161	2.975	6.057	3.082
ARNT	PAS-A	FG Loop	T243	16.66	13.424	-3.236
			V244	14.679	11.616	-3.063
		GH Loop	M279	4.547	7.703	3.156
			N280	4.101	7.168	3.067
			R281	3.533	6.721	3.188
**Holo vs Holo-p**						
Protomer	Domain	Structural Element	Residue	Holo	Holo-p	Difference
HIF-2α	PAS-A	GH Loop	N184	2.147	5.24	3.093
			L185	2.078	5.262	3.184
			K186	2.376	5.381	3.005
ARNT	PAS-A	GH Loop	L293	1.266	4.308	3.042
		HI Loop	D326	8.993	12.58	3.587
			P327	8.346	11.477	3.131

Table S3. Binding Energy decompositions of ΔG_{Total} (Total Binding Free Energy, BFE) averaged over 5 trials for holo and holo-G323E for the protein-protein binding interaction between HIF-2α and ARNT are shown for several structural components. Total average BFE for HIF-2α, ARNT, and whole dimer are shown. FG Loop, GH Loop, and HI Loop energies were included in PAS-A "Complete" energies and were not included separately in calculations of total.

				Average BFE (kJ/mol)		
Protomer	Domain	Structure	Residue(s)	Holo	Holo-G323E	Δ
HIF-2α		N-terminus	26	193.257	180.293	-12.964
	bHLH	Complete	27-73	-24.981	-8.367	16.614
		bHLH/PAS-A Linker	74-81	-132.56	-154.609	-22.049
	PAS-A	Complete	82-228	-369.329	-413.853	-44.524
		FG Loop	148-164	97.281	134.962	37.681
		GH Loop	175-188	186.375	183.614	-2.761
		HI Loop	203-218	-29.026	-36.614	-7.588
		PAS-A/PAS-B Linker	229-242	-85.662	-83.37	2.292
	PAS-B	Complete	243-343	-72.332	-87.094	-14.762
		C-terminal Linker	344-360	-106.43	-124.871	-18.441
			HIF-2α Total	-598.037	-691.871	-93.834
ARNT		N-terminus	98	10.692	5.03	-5.662
	bHLH	Complete	99-142	-523.724	-606.976	-83.252
		bHLH/PAS-A Linker	143-160	-208.376	-227.568	-19.192
	PAS-A	Complete	161-343	6.065	-20.498	-26.563
		FG Loop	226-259	-187.39	-211.905	-24.515
		GH Loop	269-302	-334.739	-341.636	-6.897
		HI Loop	316-333	348.532	376.321	27.789
		PAS-A/PAS-B Linker	344-361	64.6	66.124	1.524
	PAS-B	Complete	362-463	-28.891	-16.539	12.352
		C-terminus	464	71.426	82.719	11.293
			ARNT total	-608.208	-717.708	-109.5
			HIF-2α + ARNT total	-1206.245	-1409.579	-203.334

Table S4. Binding Energy decompositions of ΔG_{Total} (Total Binding Free Energy, BFE) averaged over 5 trials for holo and holo-p for the protein-protein binding interaction between HIF-2α and ARNT are shown for several structural components. Total average BFE for HIF-2α, ARNT, and whole dimer are shown. FG Loop, GH Loop, and HI Loop energies were included in PAS-A "Complete" energies and were not included separately in calculations of total.

				Average BFE (kJ/mol)		
Protomer	Domain	Structure	Residue(s)	Holo	Holo-p	Δ
HIF-2α		N-terminus	26	193.257	183.663	-9.594
	bHLH	Complete	27-73	-24.981	-11.089	13.892
		bHLH/PAS-A Linker	74-81	-132.560	-157.321	-24.761
	PAS-A	Complete	82-228	-369.329	-419.691	-50.362
		FG Loop	148-164	97.281	118.165	20.884
		GH Loop	175-188	186.375	165.533	-20.842
		HI Loop	203-218	-29.026	-35.785	-6.759
		PAS-A/PAS-B Linker	229-242	-85.662	-80.702	4.960
	PAS-B	Complete	243-343	-72.332	-149.615	-77.283
		C-terminal Linker	344-360	-106.430	-91.504	14.926
			HIF-2α Total	-598.037	-726.259	-128.222
ARNT		N-terminus	98	10.692	5.448	-5.244
	bHLH	Complete	99-142	-523.724	-683.737	-160.013
		bHLH/PAS-A Linker	143-160	-208.376	-231.199	-22.823
	PAS-A	Complete	161-343	6.065	17.674	11.609
		FG Loop	226-259	-187.390	-207.441	-20.051
		GH Loop	269-302	-334.739	-328.119	6.620
		HI Loop	316-333	348.532	408.751	60.219
		PAS-A/PAS-B Linker	344-361	64.600	84.128	19.528
	PAS-B	Complete	362-463	-28.891	6.976	35.867
		C-terminus	464	71.426	83.662	12.236
			ARNT total	-608.208	-717.048	-108.84
			HIF-2α + ARNT total	-1206.245	-1443.307	-237.062

Table S5. Top 20 HIF-2α residues stabilizing HIF-2α:ARNT protein-protein binding in holo-G323E compared to holo. ΔG_{Total}, ΔE_{MM}, ΔG_{Polar}, and $\Delta G_{Non\text{-}polar}$ energy components are shown. Averaged values shown are from five holo or holo-G323E replicates. In ΔG_{Total}, Δ was calculated as holo-G323E – holo.

		ΔG_{Total} (kJ/mol)			ΔE_{MM} (kJ/mol)		ΔG_{Polar} (kJ/mol)		$\Delta G_{Non\text{-}polar}$ (kJ/mol)	
	HIF-2α Residue	Holo	Holo-G323E	Δ	Holo	Holo-G323E	Holo	Holo-G323E	Holo	Holo-G323E
1	G/E323	-1.82	-42.90	-41.08	-2.43	-45.12	0.60	2.22	0.00	0.00
2	E165	-28.07	-44.99	-16.92	-145.36	-116.58	119.26	72.84	-1.97	-1.25
3	R26	193.26	180.29	-12.96	202.46	194.78	-9.11	-14.41	-0.11	-0.08
4	K53	128.01	115.63	-12.38	148.51	124.89	-20.29	-8.84	-0.22	-0.42
5	K29	112.33	100.33	-12.00	120.96	106.38	-7.94	-5.49	-0.69	-0.55
6	E216	-72.25	-84.20	-11.95	-77.59	-92.13	5.71	8.16	-0.34	-0.24
7	E348	-6.60	-18.14	-11.54	-17.68	-23.14	11.48	5.18	-0.41	-0.18
8	D84	-30.94	-41.47	-10.53	-52.86	-67.16	22.45	26.26	-0.52	-0.59
9	E145	-59.51	-68.85	-9.34	-61.54	-71.14	2.03	2.29	0.00	0.00
10	E113	-64.92	-74.17	-9.24	-93.08	-86.50	29.89	13.90	-1.73	-1.56
11	E78	-40.24	-48.93	-8.69	-35.69	-44.37	-4.03	-3.70	-0.50	-0.88
12	E141	-74.51	-83.18	-8.67	-78.12	-88.32	3.61	5.12	0.00	-0.01
13	D105	-94.31	-102.28	-7.96	-168.25	-188.84	74.75	87.78	-0.82	-1.17
14	D88	-38.85	-46.38	-7.53	-105.25	-105.45	68.04	60.66	-1.65	-1.60
15	R27	103.32	95.92	-7.40	114.09	104.70	-10.66	-8.65	-0.12	-0.14
16	D107	-86.58	-93.56	-6.98	-181.55	-193.50	95.56	100.53	-0.59	-0.61
17	E227	-57.48	-63.49	-6.01	-157.84	-151.29	101.26	88.59	-0.88	-0.77
18	D240	-39.46	-45.20	-5.74	-106.77	-100.11	69.25	56.69	-1.95	-1.77
19	E142	-57.28	-63.01	-5.72	-59.71	-65.88	2.43	2.87	-0.01	0.00
20	R58	115.65	109.93	-5.72	128.08	123.47	-11.66	-13.04	-0.78	-0.49

Table S6. Top 20 ARNT residues stabilizing HIF-2α:ARNT protein-protein binding in holo-G323E relative to holo. ΔG_{Total}, ΔE_{MM}, ΔG_{Polar}, and $\Delta G_{Non\text{-}polar}$ energy components are shown. Averaged values shown are from five holo or holo-G323E replicates. In ΔG_{Total}, Δ was calculated as holo-G323E – holo.

		ΔG_{Total} (kJ/mol)			ΔE_{MM} (kJ/mol)		ΔG_{Polar} (kJ/mol)		$\Delta G_{Non\text{-}polar}$ (kJ/mol)	
	ARNT Residue	Holo	Holo-G323E	Δ	Holo	Holo-G323E	Holo	Holo-G323E	Holo	Holo-G323E
1	R100	-91.08	-114.49	-23.41	-92.32	-116.53	1.24	2.06	0.00	-0.02
2	R102	-66.45	-87.59	-21.14	-97.05	-156.86	31.52	70.64	-0.94	-1.35
3	R101	-54.14	-73.03	-18.89	-63.07	-83.57	9.15	10.87	-0.21	-0.32
4	R254	-107.52	-125.98	-18.46	-130.45	-181.77	23.50	56.89	-0.55	-1.11
5	R261	-116.01	-132.56	-16.55	-262.88	-276.14	149.72	146.37	-2.87	-2.80
6	R260	-119.98	-135.96	-15.98	-201.90	-205.43	83.15	70.37	-1.24	-0.91
7	K313	-97.53	-113.46	-15.92	-205.34	-210.90	109.80	99.30	-2.01	-1.89
8	K220	-80.03	-95.52	-15.49	-133.80	-131.34	55.24	37.10	-1.46	-1.30
9	R266	-119.89	-135.12	-15.23	-244.92	-243.74	127.05	110.56	-2.02	-1.94
10	K165	-101.57	-116.18	-14.60	-102.80	-123.08	1.32	7.14	-0.09	-0.23
11	R342	-150.40	-164.25	-13.85	-221.86	-223.68	72.60	60.44	-1.14	-0.99
12	K128	-71.00	-84.61	-13.61	-72.30	-86.24	1.29	1.62	0.00	0.00
13	R99	-119.13	-132.15	-13.02	-193.50	-168.70	75.27	37.40	-0.90	-0.86
14	R366	-125.21	-138.21	-12.99	-165.13	-172.86	40.50	35.14	-0.58	-0.46
15	K155	-73.81	-86.44	-12.63	-120.26	-112.25	47.16	26.39	-0.72	-0.61
16	R222	-135.95	-148.11	-12.16	-165.29	-184.31	29.74	36.46	-0.36	-0.26
17	K246	-151.11	-161.80	-10.69	-280.56	-279.02	130.98	118.48	-1.50	-1.27
18	R143	-146.18	-156.81	-10.63	-275.60	-281.75	130.93	126.45	-1.51	-1.50
19	R133	-125.36	-134.66	-9.30	-145.20	-149.83	19.84	15.18	0.00	-0.02
20	K419	-80.00	-89.21	-9.21	-82.82	-91.29	2.81	2.08	0.00	0.00

Table S7. Top 20 HIF-2α residues stabilizing HIF-2α:ARNT protein-protein binding in holo-p compared to holo. ΔG_{Total}, ΔE_{MM}, ΔG_{Polar}, and $\Delta G_{Non\text{-}polar}$ energy components are shown. Averaged values shown are from five holo or holo-p replicates. In ΔG_{Total}, Δ was calculated as holo-p – holo.

		ΔG_{Total} (kJ/mol)			ΔE_{MM} (kJ/mol)		ΔG_{Polar} (kJ/mol)		$\Delta G_{Non\text{-}polar}$ (kJ/mol)	
	HIF-2α Residue	Holo	Holo-p	Δ	Holo	Holo-p	Holo	Holo-p	Holo	Holo-p
1	T324	-0.01	-93.99	-93.98	-0.07	-95.31	0.06	1.32	0.00	0.00
2	D107	-86.58	-100.47	-13.89	-181.55	-190.61	95.56	90.73	-0.59	-0.58
3	K29	112.33	100.10	-12.24	120.96	105.58	-7.94	-5.08	-0.69	-0.42
4	K186	79.77	67.76	-12.01	79.83	67.70	-0.05	0.05	0.00	0.00
5	D105	-94.31	-105.85	-11.53	-168.25	-172.03	74.75	66.95	-0.82	-0.75
6	E113	-64.92	-76.07	-11.15	-93.08	-88.93	29.89	14.38	-1.73	-1.52
7	D133	-105.22	-116.12	-10.90	-173.68	-163.69	69.05	47.95	-0.57	-0.37
8	R26	193.26	183.66	-9.59	202.46	187.11	-9.11	-3.13	-0.11	-0.31
9	E82	-33.02	-42.47	-9.45	-50.36	-49.19	18.00	7.14	-0.66	-0.43
10	E76	-43.68	-52.95	-9.27	-46.54	-55.39	3.33	2.89	-0.46	-0.45
11	D88	-38.85	-46.49	-7.64	-105.25	-110.13	68.04	65.36	-1.65	-1.72
12	D84	-30.94	-38.21	-7.27	-52.86	-55.64	22.45	17.94	-0.52	-0.50
13	E96	-56.79	-63.64	-6.85	-162.58	-149.83	107.42	87.59	-1.63	-1.42
14	R330	29.55	22.82	-6.73	29.32	22.54	0.23	0.28	0.00	0.00
15	E80	-39.14	-45.48	-6.33	-40.70	-50.49	1.93	5.28	-0.39	-0.30
16	R58	115.65	109.77	-5.89	128.08	127.78	-11.66	-17.26	-0.78	-0.75
17	K53	128.01	122.38	-5.63	148.51	136.15	-20.29	-13.50	-0.22	-0.28
18	E125	-92.17	-97.13	-4.96	-99.10	-103.35	6.96	6.24	-0.02	-0.01
19	E216	-72.25	-77.04	-4.80	-77.59	-70.46	5.71	-6.44	-0.34	-0.13
20	E78	-40.24	-44.90	-4.67	-35.69	-46.49	-4.03	2.59	-0.50	-1.01

Table S8. Top 20 ARNT residues stabilizing HIF-2α:ARNT protein-protein binding in holo-p relative to holo. ΔG_{Total}, ΔE_{MM}, ΔG_{Polar}, and $\Delta G_{Non\text{-}polar}$ energy components are shown. Averaged values shown are from five holo or holo-p replicates. In ΔG_{Total}, Δ was calculated as holo-p – holo.

		ΔG_{Total} (kJ/mol)			ΔE_{MM} (kJ/mol)		ΔG_{Polar} (kJ/mol)		$\Delta G_{Non\text{-}polar}$ (kJ/mol)	
	ARNT Residue	Holo	Holo-p	Δ	Holo	Holo-p	Holo	Holo-p	Holo	Holo-p
1	R100	-73.81	-109.62	-35.80	-120.26	-165.26	47.16	57.01	-0.72	-1.39
2	R102	-54.14	-89.79	-35.66	-63.07	-100.61	9.15	10.99	-0.21	-0.16
3	R101	-66.45	-98.77	-32.31	-97.05	-152.79	31.52	55.17	-0.94	-1.14
4	R254	-82.47	-114.57	-32.09	-84.05	-125.72	1.57	11.35	0.00	-0.19
5	R261	-125.21	-151.55	-26.34	-165.13	-188.10	40.50	37.03	-0.58	-0.51
6	R260	-116.01	-141.52	-25.51	-262.88	-275.57	149.72	136.64	-2.87	-2.58
7	K313	-110.44	-134.80	-24.36	-119.20	-143.08	8.97	8.61	-0.22	-0.33
8	K220	-119.89	-143.47	-23.58	-244.92	-265.50	127.05	124.10	-2.02	-2.06
9	R266	-150.40	-172.32	-21.92	-221.86	-231.10	72.60	59.98	-1.14	-1.22
10	K165	-151.11	-172.31	-21.20	-280.56	-290.73	130.98	119.82	-1.50	-1.41
11	R342	-146.18	-167.04	-20.86	-275.60	-288.47	130.93	123.22	-1.51	-1.79
12	K128	-40.79	-61.24	-20.45	-50.89	-63.54	10.39	2.42	-0.29	-0.12
13	R99	-80.03	-100.29	-20.27	-133.80	-116.68	55.24	16.80	-1.46	-0.38
14	R366	-97.53	-117.11	-19.57	-205.34	-207.48	109.80	92.19	-2.01	-1.82
15	K155	-135.95	-155.27	-19.33	-165.29	-185.90	29.74	30.99	-0.36	-0.36
16	R222	-133.52	-152.34	-18.82	-138.37	-157.30	4.87	4.98	-0.02	-0.02
17	K246	-91.08	-109.43	-18.35	-92.32	-113.79	1.24	4.39	0.00	-0.03
18	R143	-119.98	-137.00	-17.02	-201.90	-214.47	83.15	78.36	-1.24	-0.91
19	R133	-53.02	-69.52	-16.50	-114.29	-134.65	62.60	66.91	-1.33	-1.77
20	K419	-80.00	-96.27	-16.27	-82.82	-98.23	2.81	1.96	0.00	0.00

Table S9. Important residues distinguishing HIF-2α domain from G323E-HIF-2α domain in binding to either ARNT or another HIF-2α domain across 6 interfaces. LR = Logistic Regression, RF = Random Forest, MLP = Multilayer perceptron. ML importances > 0.8 are bolded. Residue importance category also shown, where category 1 indicates ML importance > 0.8 in one of the ML models, 2 indicates | $\Delta\Delta G_{Total}$ | ≥ 2 kJ/mol or | ΔRMSF | ≥ 1Å (≥ 3Å if disordered) and meeting criteria for category 1, and category of 3 indicates all criteria are met.

			Importance					
	Domain	Residue	LR	RF	MLP	$\Delta\Delta G_{Total}$	ΔRMSF	Residue Importance Category
Interface 1	HIF-2α bHLH	L37	**1**	0.633	0.606	-1.725	0.163	1
		R58	0.752	**1**	**1**	-5.722	0.556	2
		R65	**0.899**	0.454	**0.821**	7.186	0.211	2
	ARNT bHLH	K104	0.519	**1**	0.689	-5.585	1.3	3
		Y108	**1**	0.141	0.616	-0.44	0.092	1
		L112	**0.883**	0.255	0.54	-0.845	0.114	1
		M139	**0.927**	0.161	0.76	1.663	0.706	1
		K140	**0.863**	0.674	0.686	-0.828	0.689	1
		L142	0.408	0.794	**1**	-0.003	0.823	1
Interface 2	HIF-2α PAS-A	D84	**0.823**	0.111	**0.826**	-10.531	1.149	3
		S163	**0.878**	**1**	**0.913**	4.051	1.637	2
		E165	**0.877**	0.64	**0.941**	-16.916	0.707	2
		R200	**0.831**	0.259	0.618	2.311	0.19	2
	ARNT PAS-A	R281	**0.988**	**0.925**	**0.875**	-0.943	3.188	2
		L282	0.704	**0.887**	**0.972**	2.526	2.248	2
		F284	0.219	**0.983**	0.565	3.959	2.009	2
		L285	**0.941**	**0.822**	**0.946**	4.36	1.943	2
Interface 3	HIF-2α PAS-B	V343	**1**	**1**	**1**	-0.11	0.411	1
	ARNT PAS-A	I264	**1**	0.519	**0.925**	0.418	0.159	1
		G292	0.688	0.17	**0.981**	-0.058	0.567	1
		L293	0.683	0.099	**0.825**	-1.054	0.615	1
		G294	0.352	**1**	0.587	0.139	0.647	1
Interface 4	HIF-2α PAS-B	M252	**0.893**	0.285	0.549	1.493	-0.002	1
		A283	0.392	0.34	**0.808**	0.359	0.169	1
		E287	0.762	**0.997**	**0.988**	-2.028	1.172	3
		M289	**0.994**	0.785	0.78	-0.825	0.14	1
	ARNT PAS-B	P449	**1**	**1**	**1**	-0.024	0.322	1

Table S9. Important residues distinguishing HIF-2α domain from G323E-HIF-2α domain in binding to either ARNT or another HIF-2α domain across 6 interfaces. LR = Logistic Regression, RF = Random Forest, MLP = Multilayer perceptron. ML importances > 0.8 are bolded. Residue importance category also shown, where category 1 indicates ML importance > 0.8 in one of the ML models, 2 indicates | $\Delta\Delta G_{Total}$ | ≥ 2 kJ/mol or | ΔRMSF | ≥ 1Å (≥ 3Å if disordered) and meeting criteria for category 1, and category 3 indicates all criteria are met.

			Importance					
	Domain	Residue	LR	RF	MLP	$\Delta\Delta G_{Total}$	ΔRMSF	Residue Importance Category
Interface 5	HIF-2α PAS-B	K291	0.78	**0.997**	0.708	-4.214	1.919	3
		Q301	**1**	0.128	0.408	-0.398	0.467	1
		S304	0.711	**0.973**	**1**	0.624	0.572	1
	HIF-2α PAS-A	F169	**1**	**1**	**1**	-0.292	0.418	1
Interface 6	HIF-2α PAS-A	Q123	0.589	**0.952**	**0.993**	-0.8642	0.083	1
		V124	0.626	**0.849**	**0.803**	0.366	0.254	1
		T127	**1**	**0.902**	0.74	0.324	0.303	1
	HIF-2α bHLH	R58	0.762	**0.933**	0.646	-5.722	0.556	2
		R65	**1**	0.394	0.525	7.186	0.211	2
		K68	**0.854**	**0.973**	**1**	12.596	-0.11	2

Table S10. Important residues distinguishing HIF-2α domain from pT324-HIF-2α domain in binding to either ARNT or another HIF-2α domain across 6 interfaces. LR = Logistic Regression, RF = Random Forest, MLP = Multilayer perceptron. ML importances > 0.8 are bolded. Residue importance category also shown, where category 1 indicates ML importance > 0.8 in one of the ML models, 2 indicates | $\Delta\Delta G_{Total}$ | ≥ 2 kJ/mol or | ΔRMSF | ≥ 1Å (≥ 3Å if disordered) and meeting criteria for category 1, and category 3 indicates all criteria are met.

			Importance					
	Domain	Residue	LR	RF	MLP	$\Delta\Delta G_{Total}$	ΔRMSF	Residue Importance Category
Interface 1	HIF-2α bHLH	E30	0.300	**0.866**	0.652	12.980	2.516	3
		T31	**0.815**	**1**	**0.953**	1.058	1.422	2
		R58	**0.966**	**0.845**	**0.971**	-5.888	0.464	2
		I61	**0.915**	0.595	**0.834**	-0.024	0.475	1
	ARNT bHLH	R133	0.729	**1**	**0.966**	-16.500	0.874	2
		V136	**1**	**0.908**	**0.982**	-0.790	0.941	1
Interface 2	HIF-2α PAS-A	Q85	0.764	0.167	**0.802**	-0.745	1.182	2
		Q86	0.549	0.188	**0.898**	-0.688	0.837	1
		M87	0.525	0.634	**0.857**	0.935	0.659	1
		L90	0.307	**0.891**	**0.959**	1.427	0.044	1
		K160	**0.942**	**0.964**	**0.868**	16.886	1.678	2
		D161	0.753	0.593	0.646	4.233	1.695	2
		S163	**0.964**	0.755	0.794	1.757	0.638	1
		R200	0.631	0.500	**0.884**	4.698	0.324	2
	ARNT PAS-A	R281	0.561	**0.922**	0.568	-7.241	1.947	2
		L282	0.658	0.584	**0.814**	1.729	1.489	1
		L285	**0.995**	0.599	**0.971**	0.297	1.179	1
		R286	0.426	0.587	**0.959**	-14.952	0.428	2
		R288	**0.843**	**0.951**	0.529	-4.025	0.970	2
		A318	0.217	**0.965**	0.461	3.536	1.043	2
Interface 3	HIF-2α PAS-B	V303	0.766	**1**	0.596	1.211	0.900	1
		V343	**1**	0.399	1	0.405	0.656	1
	ARNT PAS-A	I264	0.686	**0.875**	**1**	1.178	0.393	1
		L293	**1**	0.784	**0.899**	4.667	3.042	3
		H307	0.666	**1**	0.657	-1.262	0.161	1
Interface 4	HIF-2α PAS-B	Y278	0.546	0.288	**0.844**	1.390	0.213	1
		E287	0.485	0.787	**0.984**	-3.151	0.796	2
		L296	**1**	**1**	**0.928**	-0.614	0.767	1
	ARNT PAS-B	P449	**1**	**1**	**1**	0.974	0.668	1

Table S10. Important residues distinguishing HIF-2α domain from pT324-HIF-2α domain in binding to either ARNT or another HIF-2α domain across 6 interfaces. LR = Logistic Regression, RF = Random Forest, MLP = Multilayer perceptron. ML importances > 0.8 are bolded. Residue importance category also shown, where category 1 indicates ML importance > 0.8 in one of the ML models, 2 indicates | $\Delta\Delta G_{Total}$ | ≥ 2 kJ/mol or | ΔRMSF | ≥ 1Å (≥ 3Å if disordered) and meeting criteria for category 1, and category 3 indicates all criteria are met.

			Importance					
	Domain	Residue	LR	RF	MLP	$\Delta\Delta G_{Total}$	ΔRMSF	Residue Importance Category
Interface 5	HIF-2α PAS-B	Q301	**0.835**	0.404	**0.980**	0.058	0.986	1
		V303	0.561	0.272	**0.871**	1.211	0.900	1
		T324	**0.970**	**1**	0.751	-93.983	1.055	3
	HIF-2α PAS-A	F169	0.314	0.741	**0.901**	-0.203	0.502	1
		T177	0.199	**0.972**	0.308	-0.790	0.877	1
		V192	0.713	0.689	**0.966**	-0.111	0.392	1
		H194	0.606	0.300	**0.815**	-2.299	0.273	2
		P228	**0.990**	0.291	0.788	0.343	0.183	1
Interface 6	HIF-2α PAS-A	F110	0.548	**0.937**	0.727	0.096	0.070	1
		E113	0.435	0.525	**0.957**	-11.148	0.014	2
		Q123	**0.938**	0.741	**0.895**	1.825	-0.019	1
		V124	0.538	0.762	**0.851**	0.325	0.110	1
		T127	**0.973**	**0.843**	0.796	-0.220	0.174	1
	HIF-2α bHLH	R58	0.622	**1**	**1**	-5.888	0.464	2
		R65	**1**	0.780	**0.870**	12.765	0.341	2

Table S11. List of the important residues found by Wu and colleagues to stabilize the HIF-2α:ARNT heterodimer *in vitro* and the difference in their interchain average ΔG_{Total} Binding Free Energy (BFE) contribution between either holo-G323E or holo-p and holo[28]. Information about sites studied through in vitro co-immunoprecipitation was obtained by referencing the work of Motta and colleagues[52]. ΔBFE is calculated by subtracting the holo value from either holo-G323E or holo-p. ΔBFE with absolute value at least -2 kJ/mol are bolded.

Protomer	Domain	Secondary Structure	Position	Holo-G323E $\Delta\Delta G_{Total}$ (kJ/mol)	Holo-p $\Delta\Delta G_{Total}$ (kJ/mol)
HIF-2α	PAS-A	Gβ	F169	-0.29	-0.2
		Hβ	V192	0.07	-0.11
			**H194**	-0.22	**-2.3**
	PAS-B	Hβ	**Q322**	-1.03	**-2.11**
		Iβ	M338	0.14	0.1
ARNT	PAS-A	A'α	L167	-0.17	0.44
			I168	-0.90	-0.42
			A171	-0.47	0.45
	PAS-B	Aβ	**R366**	**-15.92**	**-19.57**
		Iβ	N448	-0.75	0.68
			Y456	-1.52	0.59

Table S12. Structural water hydrogen bond networks between belzutifan (bel) and HIF-2α PAS-B drug binding domain are shown for holo (h), holo-G323 (hE), and holo-p (hp) replicates 1-5. Donor-acceptor pairs as well as occupancy (%) out of 250 equally spaced frames from last 250 ns.

State	Replicate	Water molecule	Belzutifan (bel)			HIF-2α PAS-B		
			Donor	Acceptor	%	Donor	Acceptor	%
Holo	h2	SOL30819	bel-Side-O1	Water	17.9	Water	A277-Main-O	59.8
						Y281-Side-OH	Water	75.3
						Water	H293-Side-NE2	54.6
		SOL31613	Water	bel-Side-O3	29.5	Water	Y307-Side-OH	22.7
						Water	S304-Main-O	37.1
						T321-Side-OG1	Water	28.2
	h3	SOL17301	bel-Side-O1	Water	21.9	Y281-Side-OH	Water	24.3
						Water	M289-Main-O	18.3
		SOL38503	bel-Side-O1	Water	37.1	Y281-Side-OH	Water	37.1
						Water	M289-Main-O	31.9
Holo-G323E	hE1	SOL32715	Water	bel-Side-O2	10.8	S292-Main-N	Water	11.6
	hE2	SOL39448	Water	bel-Side-O3	10.4	Water	G305-Main-O	17.9
						T321-Side-OG1	Water	16.7
	hE3	SOL31602	bel-Side-O1	Water	12	Water	A277-Main-O	27.1
			Water	bel-Side-O1	14.3	Y281-Side-OH	Water	39
		SOL31615	Water	bel-Side-N1	32.7	Water	A277-Main-O	69.3
						F280-Main-N	Water	36.3
	hE4	SOL9318	Water	bel-Side-O2	72.9	H293-Main-N	Water	17.1
						S292-Main-N	Water	51.4
		SOL32119	Water	bel-Side-O4	19.1	Water	E323-Side-OE2	29.5
						Water	E323-Side-OE1	27.1
						C339-Side-SG	Water	13.9
	hE5	SOL25734	Water	bel-Side-O4	24.7	Water	E323-Side-OE1	80.1
						Water	Q322-Main-O	23.9

Table S12. Structural water hydrogen bond networks between belzutifan (bel) and HIF-2α PAS-B drug binding domain for holo (h), holo-G323 (hE), and holo-p (hp) replicates 1-5. Donor-acceptor pairs as well as occupancy (%) out of 250 equally spaced frames from last 250 ns.

State	Replicate	Water molecule	Belzutifan (bel)			HIF-2α PAS-B		
			Donor	Acceptor	%	Donor	Acceptor	%
Holo-p	hp1	SOL2285	Water	bel-Side-O1	14.7	Water	A277-Main-O	21.5
						Y281-Side-OH	Water	31.1
		SOL20493	Water	bel-Side-O1	23.5	Water	A277-Main-O	35.9
						Y281-Side-OH	Water	50.2
		SOL31600	Water	bel-Side-N1	47.0	F280-Main-N	Water	22.3
						Water	A277-Main-O	21.5
		SOL31776	Water	bel-Side-O2	19.1	Water	S292-Main-O	19.9
						L296-Main-N	Water	19.9
	hp2	SOL27983	Water	bel-Side-O1	30.3	Water	A277-Main-O	52.2
						Y281-Side-OH	Water	64.1
		SOL31600	Water	bel-Side-N1	43.8	F280-Main-N	Water	22.7
						Water	A277-Main-O	13.9
		SOL31610	Water	bel-Side-O1	15.9	Y281-Side-OH	Water	27.1
						Water	A277-Main-O	15.5
	hp3	SOL18343	Water	bel-Side-O2	41.8	Water	S292-Main-O	43.8
						V303-Main-N	Water	79.7
						Water	V303-Main-O	17.5
		SOL29142	Water	bel-Side-O1	14.3	Y281-Side-OH	Water	77.3
			bel-Side-O1	Water	24.3	Water	A277-Main-O	50.6
		SOL31622	Water	bel-Side-N1	36.7	Water	A277-Main-O	33.5
						F280-Main-N	Water	32.7
	hp4	SOL9200	Water	bel-Side-O2	42.6	Water	V302-Main-O	36.3
						V302-Main-N	Water	45.8
		SOL13870	Water	bel-Side-O2	31.9	V302-Main-N	Water	35.5
						Water	V302-Main-O	23.9
	hp5	SOL37275	Water	bel-Side-O2	18.7	V303-Main-N	Water	17.1

Table S13. Protein-ligand Binding Free Energy (BFE) components, namely ΔE_{MM} , ΔG_{Polar} , $\Delta G_{Non\text{-}polar}$, and ΔG_{Total} are shown for both holo and holo-G323E. Top 10 residues with the highest ΔG_{Total} shown, and Δ was calculated as holo-G323E minus holo.

		ΔG_{Total}			ΔE_{MM}(kJ/mol)			ΔG_{Polar} (kJ/mol)			$\Delta G_{Non\text{-}polar}$ (kJ/mol)		
	Residues	Holo	Holo-G323E	Δ	Holo	Holo-G323E	Δ	Holo	Holo-G323E	Δ	Holo	Holo-G323E	Δ
1	G/E323	-1.047	28.594	29.641	-1.490	-14.401	-12.911	0.489	43.580	43.091	-0.047	-0.585	-0.538
2	L296	-3.964	-0.878	3.086	-4.840	-1.317	3.523	1.125	0.497	-0.629	-0.251	-0.058	0.194
3	K291	0.100	2.523	2.423	-0.656	-3.815	-3.159	0.756	6.392	5.636	0.000	-0.052	-0.052
4	H248	-0.282	2.094	2.376	-8.757	-5.216	3.541	8.911	7.684	-1.227	-0.436	-0.376	0.060
5	V302	-2.579	-0.330	2.250	-2.879	-0.875	2.004	0.412	0.552	0.140	-0.111	-0.006	0.105
6	S292	-0.555	1.245	1.800	-3.451	-3.554	-0.103	3.011	5.113	2.101	-0.115	-0.315	-0.200
7	I337	-3.350	-1.573	1.777	-3.847	-1.157	2.690	0.667	-0.318	-0.985	-0.170	-0.099	0.071
8	C339	-4.124	-2.465	1.659	-5.640	-2.610	3.030	1.897	0.290	-1.607	-0.382	-0.145	0.236
9	H293	-2.385	-0.835	1.550	-9.177	-3.910	5.267	7.136	3.313	-3.823	-0.342	-0.238	0.104
10	M252	-4.658	-3.136	1.522	-5.478	-3.789	1.689	1.065	1.073	0.008	-0.243	-0.420	-0.176

Movie S1 Movie showing tugging motion of N178 and R179 on phosphorylated T324. Last 250 ns of 5 holo-p replicates were concatenated to form the 1.25 μs trajectory shown. Only HIF-2α protomer is shown while the ARNT protomer is hidden.

Movie S2 Movie showing lack of tugging motion of N178 and R179 on unphosphorylated T324. Last 250 ns of 5 holo replicates were concatenated to form the 1.25 μs trajectory shown. Only HIF-2α protomer is shown while the ARNT protomer is hidden.
